# Pictolysin-III, a Hemorrhagic Type-III Metalloproteinase Isolated from *Bothrops pictus* (Serpentes: Viperidae) Venom, Reduces Mitochondrial Respiration and Induces Cytokine Secretion in Epithelial and Stromal Cell Lines

**DOI:** 10.3390/pharmaceutics15051533

**Published:** 2023-05-18

**Authors:** Dan E. Vivas-Ruiz, Paola Rosas, Alex Proleón, Daniel Torrejón, Fanny Lazo, Ana Belén Tenorio-Ricca, Francisco Guajardo, Cristopher Almarza, Víctor Andrades, Jessica Astorga, Daniel Oropesa, Jorge Toledo, María Jesús Vera, Jorge Martínez, Ramiro Araya-Maturana, Karen Dubois-Camacho, Marcela A. Hermoso, Valéria G. Alvarenga, Eladio Flores Sanchez, Armando Yarlequé, Luciana Souza Oliveira, Félix A. Urra

**Affiliations:** 1 Laboratorio de Biología Molecular, Facultad de Ciencias Biológicas, Universidad Nacional Mayor de San Marcos, Av. Venezuela Cdra 34 S/N, Ciudad Universitaria, Lima Cercado, Lima 15081, Peru; 2 Network for Snake Venom Research and Drug Discovery, Av. Independencia 1027, Santiago 7810000, Chile; 3 MIBI: Interdisciplinary Group on Mitochondrial Targeting and Bioenergetics, Universidad de Talca, Talca 3460000, Chile; 4 Metabolic Plasticity and Bioenergetics Laboratory, Molecular and Clinical Pharmacology Program, Institute of Biomedical Sciences, Faculty of Medicine, University of Chile, Av. Independencia 1027, Santiago 7810000, Chile; 5 Advanced Scientific Equipment Network (REDECA), Faculty of Medicine, Universidad de Chile, Santiago 8380453, Chile; 6 Laboratorio de Biología Celular, INTA, University of Chile, Santiago 7810000, Chile; 7 Instituto de Química de Recursos Naturales, Universidad de Talca, Talca 3460000, Chile; 8 Laboratory of Innate Immunity, Program of Immunology, Institute of Biomedical Sciences, Faculty of Medicine, University of Chile, Santiago 7810000, Chile; 9 Department of Gastroenterology and Hepatology, University Medical Center Groningen, 9713 Groningen, The Netherlands; 10 Laboratory of Biochemistry of Proteins from Animal Venoms, Research and Development Center, Ezequiel Dias Foundation, Belo Horizonte 30510-010, Brazil

**Keywords:** snake venoms, mitochondrial bioenergetics, oxidative phosphorylation, GPVI antagonist, BH3 mimetics, Venetoclax

## Abstract

From the venom of the *Bothrops pictus* snake, an endemic species from Peru, we recently have described toxins that inhibited platelet aggregation and cancer cell migration. In this work, we characterize a novel P-III class snake venom metalloproteinase, called pictolysin-III (Pic-III). It is a 62 kDa proteinase that hydrolyzes dimethyl casein, azocasein, gelatin, fibrinogen, and fibrin. The cations Mg^2+^ and Ca^2+^ enhanced its enzymatic activity, whereas Zn^2+^ inhibited it. In addition, EDTA and marimastat were also effective inhibitors. The amino acid sequence deduced from cDNA shows a multidomain structure that includes a proprotein, metalloproteinase, disintegrin-like, and cysteine-rich domains. Additionally, Pic-III reduces the convulxin- and thrombin-stimulated platelet aggregation and in vivo, it has hemorrhagic activity (DHM = 0.3 µg). In epithelial cell lines (MDA-MB-231 and Caco-2) and RMF-621 fibroblast, it triggers morphological changes that are accompanied by a decrease in mitochondrial respiration, glycolysis, and ATP levels, and an increase in NAD(P)H, mitochondrial ROS, and cytokine secretion. Moreover, Pic-III sensitizes to the cytotoxic BH3 mimetic drug ABT-199 (Venetoclax) in MDA-MB-231 cells. To our knowledge, Pic-III is the first SVMP reported with action on mitochondrial bioenergetics and may offer novel opportunities for promising lead compounds that inhibit platelet aggregation or ECM–cancer-cell interactions.

## 1. Introduction

Snake venoms are a natural source of active proteins, also known as toxins, with enzymatic (metalloproteinases, serineproteinases, phospholipases A2, and L-amino oxidases) or non-enzymatic (neurotoxins, disintegrins, C-type lectins, cytotoxins, myotoxins, and cardiotoxins) activities. Several of these toxins are multifunctional and may act on multiple protein targets of their preys, which are highly specific to disrupt several biological processes in human cells, including cancer cells [1,2,3,4]. A plethora of studies on human envenoming has shown the functional diversity of snake toxins [1,5]; however, molecular mechanisms of action of several of these biomolecules remain poorly studied.

A particular relevance corresponds to the snake venom metalloproteinases (SVMPs), which represent more than 30% of most viperid and crotalid venoms [6]. Together with the mammalian ADAM (a disintegrin and metalloproteinase) family, the SVMPs are members of the reprolysin subfamily of metalloproteinases [7]. They are divided into three groups (P-I, P-II, and P-III) depending on their domain structure. The P-I proteinases (20–30 kDa) have only the catalytic metalloprotease domain (M) and can be hemorrhagic or non-hemorrhagic [8]. The P-II class has in addition to the M domain, a C-terminal disintegrin (D) domain. Several disintegrins have the [R/KGD] motif that mediates the interaction with integrins. Thus, an important effect is the inhibition of platelet aggregation through interaction with the integrin αIIbβ3 [9]. The P-III toxins (60–100 kDa) present the greatest complexity within the SVMPs, exhibiting the M and D domains (with mainly the ECD motif) plus a domain rich in cysteine (C) whose main role is the recognition of and binding to substrates [10]. Additionally, P-IV toxins are heterotrimeric and possess an additional snake C-type lectin-like (snaclec) domain, which are now included in the P-III group [8].

The SVMPs act on cell–cell and extracellular matrix (ECM) interactions [11] through the proteolytic degradation of collagen type I and type IV, fibronectin, laminin, nidogen, vitronectin, and other basement membranes, ECM, and plasma components of the vascular endothelium [11,12,13,14,15,16]. As a result of this action, they induce local and systemic hemorrhage, the principal pathophysiological effect of SVMPs [17,18]. Some SVMPs also produce intravascular coagulation, edema, inflammation, necrosis, fibrin(ogen) degradation, induction of apoptosis, and inhibition of platelet aggregation [8,17,19,20]. Recently, a study using crude *Macrovipera lebetina obtusa* (*Levantine viper*) venom suggested that possibly SVMPs may disrupt the mitochondrial metabolism in the Vero monkey kidney epithelial cell line [21]. To our knowledge, there are no studies that identify the action of an isolated SVMP on mitochondrial respiration and its implication for the disruptive action of the cell–ECM interaction.

In migrating cancer cells, the ECM composition and stiffness are drivers for metabolic shifts toward enhanced mitochondrial respiration and local mitochondrial accumulation in the leading-edge lamellipodia [22]. Notably, some SVMPs inhibit angiogenesis, cell viability, adhesion, and migration in cancer cell lines by disruption of ECM–cell interactions [2,4,23,24,25,26]; however, the action of these anti-cancer SVMPs on cellular metabolism and mitochondrial bioenergetics remains completely unknown. This fact postulates SVMPs as interesting prototypes for the development of new anti-cancer drugs [2,4].

Our team has previously reported the presence of Bpic-Laao (an L-amino acid oxidase) and pictobin (a thrombin-like enzyme) in the *Bothrops pictus* venom, an endemic species from Peru of medical relevance due to cases of ophidism [27,28]. These toxins interfere with platelet aggregation, as well as cell survival, migration, and mitochondrial bioenergetics of breast cancer cell lines [28]. In the present work, we described the biochemical, molecular, and anti-cancer characteristics of pictolysin-III (Pic-III), the first P-III SVMP reported with inhibitory effects on mitochondrial bioenergetics.

## 2. Materials and Methods

### 2.1. Chemical and Reagents

All reagents were obtained from Sigma-Aldrich Corp. (St. Louis, MO, USA). Polyvalent antibothropic serum (Batch: 01000376) was obtained from the Instituto Nacional de Salud (INS, Peru).

### 2.2. Venom

*Bothrops pictus* venom was collected from four adult specimens (three males and one female) captured within the district of Pachacamac (southern Lima, Peru), and kept in captivity at the Serpentarium “Oswaldo Meneses”—Museo de Historia Natural, Universidad Nacional Mayor de San Marcos (UNMSM), Lima, Peru. Venoms were obtained by mechanical pressure on the venom glands and collected in free-DNAase and RNAse sterile tubes. The venom pool was lyophilized and stored at 4 °C or mixed with RNA Later for RNA purification. The handling of live snakes for extraction of venom was approved by the ethics committee of FCB-UNMSM (Code N°: 014-2022-CBE-FCB-UNMSM).

### 2.3. Purification of Pictolysin-III

*Bothrops pictus* venom (682 mg) was dissolved in 4.5 mL 50 mM ammonium acetate buffer (pH 7.4, 0.3 M NaCl), centrifuged at 6000× *g*, and the supernatant was loaded on Sephacryl S-200 resin packed in tandem of two (2.5 cm × 100 cm each) columns in the same buffer, and eluted at a flow rate 13 mL/h. Peak 1 containing proteolytic and hemorrhagic activities (155 mg) was pooled, dialyzed against a 1 mM CaCl_2_ solution, and lyophilized. This fraction was dissolved in 3.5 mL 50 mM Tris buffer (pH 8.5 containing 2 mM CaCl_2_) and applied on a DEAE-Sepharose CL-6B (1.6 cm × 17.5 cm) column with a linear salt gradient from 0–0.3 M NaCl at a flow rate of 11 mL/h. Proteolytically active and hemorrhagic fractions (peak C, 31 mg) were pooled, dialyzed against a 1 mM CaCl_2_ solution, and lyophilized. Finally, this fraction was dissolved in 20 mM Hepes buffer (pH 7.0, containing 1 mM CaCl_2_) and separated by a CM Sepharose CL 6B (1.5 cm × 22 cm) column with a linear salt gradient from 0–0.3 M NaCl at a flow rate of 13 mL/h. The proteolytically active and hemorrhagic fractions (17.9 mg) containing Pic-III were pooled and dialyzed against a 1 mM CaCl_2_ solution, distilled, and lyophilized. To separate the native protein from the peptide products of autoproteolysis, an MLPC (Biobase) chromatography system was employed using an Enrich SEC 70 10 × 300 size exclusion column at a flow rate of 1.0 mL/min. Uncropped gels are shown in the Supplementary Information.

### 2.4. Western Blotting

Peak 1 and peak 2 (10 μg) from the CM-Sepharose step were submitted to 12% SDS-PAGE, and the gel bands were transferred onto a nitrocellulose membrane (0.22 μm pore size) for 18 h at 30 V at 4 °C. Subsequently, the membrane was blocked with a blocking buffer (5% skimmed milk, 0.15 M NaCl, 0.05% tween-20, 0.02 M Tris-HCl, pH 7.5) for 2 h, with agitation at room temperature. Then, the membrane was washed three times with washing buffer and incubated with anti-Atr-III serum (provided by FUNED, Brazil; Atr-III: a P-III SVMP from *B. atrox*) (dilution 1:1000 in blocking buffer) for 2 h at room temperature. After incubation, three more washes were conducted and the membrane was incubated with anti-rabbit IgG-horseradish peroxidase (Sigma-Aldrich, diluted 1:5000 in blocking buffer) for 1 h at room temperature. Finally, after another three washes, the membrane was revealed with ECL reagent (Thermo Scientific, Waltham, MA, USA). The image was acquired using Chemidoc (Bio-Rad, Hercules, CA, USA). The uncropped Western blot is shown in the Supplementary Information.

### 2.5. Molecular Characterization

#### 2.5.1. cDNA-Encoding Pictolysin-III and Gene Expression Analysis

Total RNA was obtained according to Vivas-Ruiz et al. [29]. The gene of Pic-III was amplified with the kit Master Mix Platinum^®^ Taq DNA Polymerase (Invitrogen, Carlsbad, CA, USA) according to the manufacturer’s instructions using the external primers MpIIIF and MpIIIR and the internal primers MpIIIFi and MpIIIRi provided by Oliveira et al. [30]. The Pic-III gene expression level was analyzed according to Vivas-Ruiz et al. [28] using Verso 1-Step qPCR ROX Kit (ThermoFisher Scientific, Waltham, MA, USA). The master mix was prepared following the manufacturer’s instructions. The assay was conducted in a 7500 Applied Biosystem Thermocycler (ThermoFisher Scientific, USA). Pictobin, Bpic-LAAO, and phospholipase A2 (PLA2) genes were used for comparison, and GAPDH was used as a housekeeping gene.

#### 2.5.2. Cloning and Nucleotide Sequencing

The cDNA transcript encoding Pic-III was inserted into the pCR2.1-TOPO vector following the TOPO-TA cloning strategy and transformed into One Shot™ *Escherichia coli* TOP10 competent cells, according to the manufacturer’s recommended protocol. Transformed *E. coli* cells were grown on Luria–Bertani (LB) agar plates overnight at 37 °C and spiked with ampicillin (50 µg/mL) for selection. Positive colonies were selected from agar plates by colony PCR amplification using M13 primers. Bacterial glycerol stocks were prepared for long-term storage at −80 °C. The identity of the Pic-III-encoding transcript was confirmed by sequencing on an ABI 3730 XL automated sequencer (Macrogen, Inc., Seoul, Republic of Korea).

#### 2.5.3. In Silico Protein Analysis

The amino acid sequence of Pic-III was obtained from cDNA using the ORF finder program [31] and analyzed with NCBI BLAST [32]. The presence of a signal peptide and protein domains was performed on HMMER [33] and PROSITE [34] servers. The tertiary structure (excluding peptide signal and pro-peptide sequence) was built by homology modeling using the MODELLER program v.10.0 [35] employing as a template catrocollastatin (P-III SVMP from *Crotalus atrox*, PDB: 2DW0), due to its high similarity (88.31%) and a high degree of resolution (2.1 Å). The three-dimensional structure was refined with GalaxiRefine server [36] and visualized with Pymol v.2.5. The model quality assessment was performed with PROCHECK [37] and for Ramachandran plot analysis, we used the SAVES tool (https://saves.mbi.ucla.edu/ accessed on 26 August 2022).

#### 2.5.4. Sequence-Based Analysis and Phylogenetic Analysis

The theoretical isoelectric point and molecular mass were estimated with the ExPASy ProtParam tool [38]. Multiple sequence alignment of Pic-III with atroxlysin-III (AQS99160), catrocollastatin (AAC59672.1), P-III SVMP from *Bothrops jararaca* (KAG5858165.1), and ADAM 12 from *Homo sapiens* (AAC08702.2) was performed by ClustalX 2.0 [39]. The Pic-III architecture was recognized based on alignment with catrocollastatin [40]. On the other hand, nucleotide sequences of P-III SVMP obtained from NCBI (https://www.ncbi.nlm.nih.gov/protein/ accessed on 27 August 2022) were aligned by ClustalX 2.0 and a phylogenetic tree was built using the maximum likelihood statistical method in the MEGA X program [41] with the Bootstrap method (2000 replications) and the General Time Reversible model with invariant sites (GTR + I).

### 2.6. Biochemical Characterization

#### 2.6.1. Enzymatic Activity

Azocasein was used as a substrate for the enzymatic characterization of Pic-III according to Gomes et al. [42], with some modifications: azocasein (1.5 mg/mL) in 20 mM Tris-HCl, 5 mM CaCl_2_ was incubated with 5 µL of Pic-III (1 mg/mL) at 37 °C. After, 45 µL of trichloroacetic acid 20% (*w*/*v*) was added to stop the reaction. Microtubes were incubated at room temperature for 20 min, centrifuged at 5000× *g* for 15 min, and, finally, the supernatant was transferred to a 96-well plate. The absorbance of the supernatant was determined at 405 nm, using the ER-500 microplate reader (Sinnowa, Nanjing, China). The increase of 0.01 absorbance units at 405 nm was defined as one unit of enzymatic activity.

#### 2.6.2. Interaction of Pictolysin-III with a TLE (Pictobin) and Plasma Inhibitor α2-Macroglobulin (α2-M)

Pic-III was incubated alone or with Pictobin (a thrombin-like enzyme from *B. pictus*) and/or α2-M (1:1:1 molar ratio) in 0.2 M Hepes buffer, pH 7.4 containing 0.15 M NaCl at 37 °C for 30 min. After that, 5 μL of the mixture was used to test the proteolytic activity using azocasein as a substrate following the method above mentioned.

#### 2.6.3. Effect of pH, Temperature, and Inhibitors

To evaluate the effect of temperature, Pic- III (1 mg/mL) was pre-incubated for 30 min at 5 °C, 25 °C, 35 °C, 45 °C, 55 °C, and 75 °C. The effect of pH on enzymatic activity was evaluated using buffers at 4.0, 5.0, 6.0, 7.0, 8.0, 9.0, and 10.0; 10 mM [c.f.] cations (CaCl_2_, MgCl_2_, and ZnCl_2_); and 10 mM inhibitors (DTT, EDTA, PMSF, 2β-mercaptoethanol, and marimastat). Additionlly, Pic-III’s azocaseinolytic activity neutralization by antibotropic polyvalent serum (INS-Peru) was evaluated by pre-incubating the enzyme with half, one, and two neutralizing doses (one neutralizing dose: 10 mL of antivenom neutralizes not less than 25 mg of *B. atrox* venom). All assays were made in triplicate.

#### 2.6.4. Effect of Pictolysin-III Deglycosylation under no Reducing Conditions on Enzymatic Activity

To maintain the functional characteristics of the enzyme, Pic-III (50 μg) was incubated with 5 μL of recombinant PNGase F (500 U) in a mixture of 20 μL of 50 mM sodium phosphate buffer (pH 7.5) and 50 μL of H_2_O MilliQ. The incubation was carried out at 37 °C for 48 h. Subsequently, 5 μL of PNGase F (500 U) was added and incubated for an additional 48 h. Finally, the mixture was applied to an MLPC column (conditions above mentioned) to obtain homogenous Pic-III. The deglycosylated enzyme was evaluated for its azocaseinolytic activity in comparison with the native Pic-III (non-PNGase treated).

#### 2.6.5. Proteolytic Activity upon Fibrinogen and Fibrin

Proteolytic activities were determined according to Oliveira et al. [30]. Human fibrinogen (H-Fg) was dissolved in 25 mM Tris-HCl, pH 7.4, 154 mM NaCl; samples with 100 µL of H-Fg (2.5 mg/mL) were incubated with 1 µg of Pic-III at 37 °C for 30 min; the reaction was stopped by adding 40 µL of denaturant buffer (SDS, β-mercaptoethanol, glycerol, and bromophenol blue). For fibrinolytic activity, fibrin was obtained using 2 NIH of human thrombin (Sigma-Aldrich) and 100 µL of H-Fg (2.5 mg/mL), incubated for 2 h at room temperature, then 1 µg of Pic-III was added to fibrin and incubated at 37 °C for 30 min. The reaction was stopped using a denaturant buffer. In both activities, the effects were observed by SDS-PAGE (14%).

### 2.7. Biological Activities

#### 2.7.1. Platelet Aggregation Assay

Human blood from two healthy volunteers (age/sex: 22/M and 23/F) was collected in acid–citrate–dextrose (ACD: 78 mM citric acid; 117 mM sodium citrate; 282 mM dextrose) [6:1, (*v*/*v*)], centrifuged at 200× *g* for 15 min to obtain platelet-rich plasma (PRP). Washed platelets were prepared as previously described by Oliveira et al. [30]. Platelet density was adjusted to 2.5 × 10^5^ platelets/µL. Washed platelets (225 µL) were pre-incubated with Pic-III (from 2 to 32 µg) in Tyrode’s solution pH 7.4, for 3 min before the addition of agonists: 6 µg/mL of convulxin (CVX) isolated from *Crotalus durissus terrificus* venom, 10 µg/mL of collagen-I, 1 U/mL thrombin or 5.5 µg/mL von Willebrand factor plus 0.5 mg/mL ristocetin. Platelet aggregation was conducted in a platelet aggregometer (AggRam Helena Laboratories, Beaumont, TX, USA) with stirring (600 rpm) at 37 °C. Light transmittance was recorded and the inhibition of platelet aggregation was measured at the maximum aggregation response.

#### 2.7.2. Hemorrhagic Activity

The hemorrhagic activity of fractions (5 μg), Pic-III, and the crude venom of *B. pictus* was analyzed by the skin assay procedure [43]. The assay was modified by using *Swiss Webster* mice. Each animal was inoculated intradermally. After 3 h, the dorsal skin was removed, and the area of the hemorrhagic halo was measured. PBS was used as a negative control. To determine the minimum hemorrhagic dose (MHD), three animals were used for each dose of Pic-III (0.5, 1.0, 2.5, 5.0, and 10.0 μg); crude venom (0.5, 0.8, 1.0, 1.5, and 4.0 μg), and PBS. The MHD corresponds to the dose of protein or venom that induced a hemorrhagic spot of 10 mm diameter and was calculated by extrapolation. This in vivo experiment was performed following the guidelines of the Brazilian College for Animal Experimentation and approved by the local Ethics Committee (Protocol number CEUA/Funed: 015/2019).

### 2.8. Studies in Cell Lines

#### 2.8.1. Cell Culture Conditions

Cell lines MDA-MB-231 (triple-negative breast cancer) and Caco-2 (colon epithelial) were purchased from the American Type Culture Collection (ATCC, Manassas, VA, USA). RMF-621, corresponds to hTERT-immortalized mammary fibroblasts derived from a reduction mammoplasty obtained via a generous gift from Dr. Charlotte Kuperwasser (Tufts University, Boston, MA, USA). RMF-621 and MDA-MB-231 were grown in Dulbecco’s modified Eagle’s medium (DMEM), containing 25 mM glucose and 4 mM glutamine supplemented with 10% fetal bovine serum (FBS), penicillin (100 IU/mL), and streptomycin (100 μg/mL). Caco-2 cells were grown in Dulbecco’s modified Eagle’s Medium/Nutrient Mixture F-12 (D-MEM/F-12) supplemented with 5% fetal bovine serum (FBS), penicillin (100 IU/mL), streptomycin (100 μg/mL), and nonessential amino acids solution 1% (GIBCO, Thermo Fisher Scientific, Grand Island, NE, USA). The culture media contained no exogenous pyruvate supplementation and cells were maintained in a humidified atmosphere at 37 °C and 5% CO_2_.

#### 2.8.2. MTT Assay

The cell viability was determined using the MTT assay. Cells were incubated in 96-well plates at 7000 cells per well and incubated for 24 h. The cells were treated with Pic-III (1, 2.5, 5, 10, 20, and 50 μg/mL in PBS) for 48 h. After treatment, the culture medium was removed, and the cells were incubated with MTT for 1 h at 37 °C. Finally, 100 μL of DMSO was added and measured by spectrophotometry at 570 nm, as described by Córdova-Delgado et al. [44].

#### 2.8.3. Determination of ATP and Mitochondrial ROS (mtROS) Levels

ATP levels were determined using a luciferin–luciferase assay system of the CellTiter-Glo Luminescent Cell Viability Assay kit (Promega, Madison, WI, USA) according to Urra et al. [45]. Cells (7000 cells/well) were incubated in 96-well plates for 24 h. The cells were treated with Pic-III (1, 2.5, 5, 10, 20, and 50 μg/mL) for 48 h. After treatment, the culture medium was removed, and then intracellular ATP levels were determined in lysed cells using a Varioskan Flash microplate reader (Thermo Scientific, USA) as described Córdova-Delgado et al. [44]. The mtROS levels were measured using MitoSOX^®^ Red Mitochondrial Superoxide Indicator (Invitrogen, Carlsbad, CA, USA). RMF-621 cells (10,000 cells/well) were incubated in 12-well plates for 24 h. The cells were treated with Vehicle (PBS) and Pic-III (50 μg/mL) for 48 h. Next, cells were incubated with MitoSOX Red^®^ (5 µM) for 30 min. Then, they were recollected, washed, and the fluorescence was detected by flow cytometry [45].

#### 2.8.4. Determination of NAD(P)H Levels

Intracellular NAD(P)H levels were measured through auto-fluorescence as previously described [46]. In brief, MDA-MB-231, Caco-2, and RMF-621 cells (5 × 10^5^ cells/mL) were seeded in 96-well plates and incubated in 100 μL PBS in the absence (Control) or presence of Pic-III (50 µg/mL), antimycin A (5 µM), or FCCP (5 µM) for 20 min. Fluorescence was measured using an excitation wavelength of 340 nm and an emission wavelength of 428 nm at 37 °C in a Synergy H1 multimode reader (Agilent, Santa Clara, CA, USA).

#### 2.8.5. Extracellular Acidification Rate in Real Time

To analyze extracellular acidification rate (ECAR) in cell lines, MDA-MB-231, Caco-2, and RMF-621 (10,000 cells/well) were seeded on XFe96 V3-PS multi-well plates and kept for 48 h at 37 °C in 5% CO_2_ with DMEM culture medium supplemented with FBS. After 48 h, cells were stimulated by 10, 20, and 50 μg/mL Pic-III for 8 h, and then the culture medium was replaced with assay media (unbuffered DMEM without red phenol, with 4 mM glutamine, and 10 mM glucose, pH = 7.4) 1 h before the assay. Glycolysis was evaluated by the sequential injection of 10 mM glucose, 1 µM oligomycin, and 100 mM 2-desoxi-D-glucose (2-DG), and ECAR was analyzed in real-time in the Agilent Seahorse XFe96 Analyzer (Seahorse Agilent, Santa Clara, CA, USA) with specific excitation and emission wavelengths of protons (470/530 nm) [45,47]. Each experiment was run at least in triplicate.

#### 2.8.6. Oxygen Consumption Rate in Real Time

MDA-MB-231, Caco-2, and RMF-621, (10,000 cells/well) were seeded on XFe96 V3-PS multi-well plates and kept for 48 h at 37 °C in 5% CO_2_ with DMEM culture medium supplemented with FBS. After 48 h, cells were stimulated by Pic-III (10, 20, and 50 μg/mL) for 8 h, and then the culture medium was replaced with assay medium (unbuffered DMEM without red phenol, with 4 mM glutamine, and 1 mM glucose, pH = 7.4) 1 h before the assay. Mitochondrial function was evaluated by the sequential injection of 1 μM oligomycin, 50 nM FCCP (MDA-MB-231), 250 nM FCCP (Caco-2), 500 nM FCCP (RMF-621), 1 μM rotenone, and 1 μM antimycin A [45,47]. The oxygen consumption rate (OCR) measurements were made with the specific excitation and emission wavelengths of the fluorescent probes for oxygen (532/650 nm). The analysis was performed in the Agilent seahorse XFe96 Analyzer. (Seahorse Agilent, Santa Clara, CA, USA). Each experiment was performed at least in triplicate.

#### 2.8.7. Caco-2 Morphology Analysis

For microscopy analysis, Caco-2 cells (4000 cells/well) were grown in Lab-Tek chambers (Thermo Fisher Scientific, USA) and cells were stimulated with 10, 20, and 50 μg/mL Pic-III for 3 h at 37 °C in a 5% CO_2_ atmosphere. At the end of each incubation period, Caco-2 cells were washed and fixed with 4% paraformaldehyde. Fixed cells were mounted with DAPI (Thermo Fisher Scientific, USA), CellMask Deep Red (Thermo Fisher Scientific, USA), and rhodamine phalloidin (Abcam, Waltham, MA, USA). Images were acquired using Cell observer Z1 (Zeiss, Oberkochen, Germany) with a 20× objective (NA 0.4, LD Plan-NEOFLUAR), with a Colibri 2 LED light source (365 nm, 470 nm, 555 nm, 590 nm), and an AxioCam MRM 12-bit CCD camera (1388 × 1040 pixels; 200 × 200 nm pixel), using the AxioVis40 × 64 V4.9.1.0 software to control the microscope. A total of 15–20 images for each experimental condition were assessed from three independent experiments using the ImageJ software (National Institutes of Health, Bethesda, MD, USA).

Automated image analysis was performed in Fiji using custom-written macros (NIH, Bethesda, MD, USA, http://rsb.info.nih.gov/ij accessed on 26 August 2022). In brief, images were equalized to the full 16-bit range, and local contrast enhancement and median filter were applied. Binary masks of approximate cell outlines were generated by the Otsu algorithm, binary operations to smoothen outlines and fill holes, and water-shedding to separate touching cells. Binary masks of cytoplasm were determined by band-pass filtering of raw cell mask images, followed by splitting of touching cells using Voronoi diagrams from binary DAPI segmentation images.

For determining the morphological characteristics of a single cell, a batch process was performed on a set of experimental images obtaining area, aspect ratio, length, and circularity using the MorphoLibJ-plugin (Plugins-MorphoLibJ-Label Images) [48,49].

#### 2.8.8. Quantitative PCR

Total RNA was isolated with Trizol (Ambion, Carlsbad, CA, USA) from RMF-621 cells treated with 20 μg/mL Pic-III for 8 h, according to the manufacturer’s instructions. Reverse transcription to complementary DNA was performed with 1 μg of RNA from each sample using M-MLV reverse transcriptase and oligo-dT (Promega, Madison, WI, USA) as a primer, according to the manufacturer’s protocol. GAPDH, IL1β, and TNFα messenger RNA (mRNA) expression was assessed by real-time PCR using a Light Cycler instrument (Roche, USA). The reaction was performed using 100 ng of complementary DNA and LightCycler1 FastStart DNA Master SYBR Green I kit (Roche, Indianapolis, IN, USA) in a final volume of 10 μL. All the reactions were performed in duplicate and negative controls were included. The sequences of the primers were as follows:

GAPDH (forward): 5′-TTG CCA TCA ATG ACC CCT TC-3′
GAPDH (reverse): 5′-ATC ATC AGC AAT GCC TCC TG-3′
IL1β (forward): 5′-AAT CCC CAG CCC TTT TGT TG-3′
IL1β (reverse): 5′-GTA AGC TAT GGC CCA CTC CA-3′
TNFα (forward): 5′-CCTGGTATGAGCCCATCTATCTG-3′
TNFα (reverse): 5′-GCAATGATCCCAAAGTAGACCTG-3′

#### 2.8.9. Cytometric Bead Array (CBA)

Cytokine secretion (TNFα, IL-8, IL-10, and IL-1β) by Caco-2 and RMF-621 cells (7.000 cells/well) treated with PBS (vehicle) or non-cytotoxic concentrations of Pic-III (Caco-2: 50 μg/mL and RMF-621: 20 μg/mL) was detected at 48 h using a Human Inflammatory Cytokines Kit (BD Biosciences, San Jose, CA, USA) following the manufacturer’s instructions. Briefly, a supernatant of cells exposed to treatments was incubated with a mixture of anti-cytokine capture antibodies-conjugated beads and PE-conjugated detector antibodies for 3 h at room temperature in the dark, subsequently washed with 1× wash buffer, and centrifuged at 200× *g* for 5 min at room temperature. Data was acquired using a FACS CantoTM II flow cytometer (BD) and analyzed using BD Cytometric Bead Array software (Version 1.4) (BD) as described [50].

### 2.9. Statistical Analysis

All statistical analyses were performed using GraphPad Prism 5.0 (GraphPad Software, San Diego, CA, USA). Statistical analysis was performed using one-way ANOVA with Bonferroni’s post-test for pairwise comparisons. The data were considered statistically significant when *p* < 0.05.

## 3. Results

### 3.1. Pictolysin-III Purification and Autoproteolysis Identification

**Figure 1:**
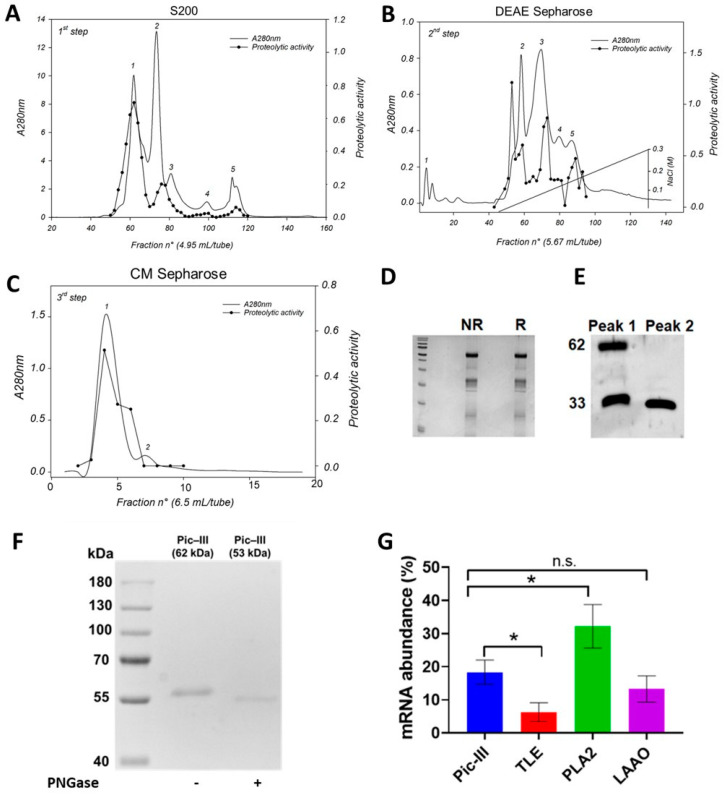
Pictolysin-III purification from *B. pictus* venom. Pic-III was purified by a three-step purification procedure as described in the Section 2. (**A**) *B. pictus* venom (682 mg) was separated on Sephacryl S-200 resin. The fractions containing proteolytic and hemorrhagic activities were pooled for the next step. (**B**) Thereafter, 155 mg of lyophilized product was applied on a DEAE-Sepharose CL-6B column with a linear salt gradient from 0–0.3 M NaCl. (**C**) Active fractions (31 mg) were pooled and applied on a CM Sepharose CL-6B column. Active metalloproteinase fractions containing Pic-III (peak 1) were pooled, dialyzed against 1 mM CaCl_2_ solution in water, and lyophilized. (**D**) The SDS-PAGE (12% gel) of purified Pic-III (5 μg) under non-reducing (NR) and reducing (R) conditions. (**E**) Peaks 1 and 2 of CM Sepharose were analyzed by Western blot using anti–Atr-III as the primary antibody. (**F**) SDS-PAGE of purified Pic-III under treatment with PNGase (+). (**G**) Transcript levels of Pic-III, TLE, PLA2, and LAAO from the venom of *B. pictus*. The levels of gene expression were analyzed by qRT-PCR real-time, using the housekeeping GAPDH. The data are presented as mean values ± SD, N = 3; TLE: thrombin-like enzyme; Pictobin; PLA2: Phospholipase A2; LAAO: L-amino acid oxidase from *B. pictus* venom. In (**G**), the data shown are the mean ± SD of three independent experiments. * *p* < 0.05 vs. Pic-III; n.s., not significant.

**Figure 2:**
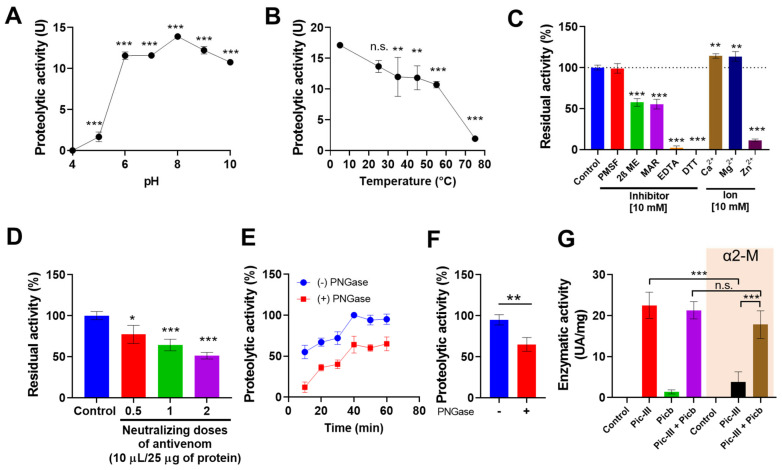
Biochemical characterization of Pictolysin-III. (**A**) Effect of pH on the proteolytic activity of Pic-III (1 mg/mL) using azocasein as the substrate. The enzyme was pre-incubated with different buffers before the determination of azocaseinolytic activity. (**B**) Effect of different temperatures on the proteolytic activity of Pic-III (1 mg/mL). (**C**) Effect of inhibitors and cations on Pic-III activity. (**D**) Inhibition of the proteolytic activity of Pic-III by PAS (INS-Peru). A total of half, one, and two neutralizing doses were tested. The enzyme (25 μg) was incubated with PAS for 30 min at 37 °C before the activity. The effect was described as % residual activity. (**E**) Pic-III (1 mg/mL), in the presence or absence of PNGase F (100 U), was incubated with azocasein to assess its proteolytic activity. (**F**) Effect of PNGase F on Pic-III proteolytic activity at 1 h of incubation. (**G**) Interaction between Pic-III and Pictobin (Picb) (1:1:1 molar ratio) with the endogenous α2-M inhibitor. Pic-III is not inhibited when it interacts with the serine protease, demonstrating a possible functional synergy. The data shown are the mean ± SD of three independent experiments. * *p* < 0.05, ** *p* < 0.01, *** *p* < 0.001 vs. control (Pic-III); n.s., not significant. In (**A**,**B**), vs. proteolytic activity at pH = 4 and 5 °C, respectively.

The enzyme was purified to homogeneity using successive chromatography on Sephacryl S-200, DEAE Sepharose CL6B, and CM Sepharose CL-6B columns, as shown in Figure 1. First, the venom (682 mg) was separated into five peaks (P1 to P5) by the S-200 column at pH 8.0 (Figure 1A). Proteolytic fractions were concentrated in P1 and between P2 and P3. These fractions were dialyzed against distilled water and lyophilized (155 mg). For the second step, the active pool was applied to a DEAE Sepharose CL-6B column with a linear salt gradient from 0–0.3 M NaCl, and five main peaks (P1 to P5) were obtained (Figure 2B). The enzymatic activity was detected in three peaks that correspond to P-III, P-II, and P-I, respectively. Thus, the enzyme corresponding fractions (before peak 2) were pooled (31 mg). In the third step, the pool from the preceding step was applied to a CM Sepharose CL-6B column (Figure 2C); by this procedure, 20 mg of the active enzyme was obtained, which was termed Pictolysin-III (Pic-III). A peculiar result was observed in this third purification step since two peaks were detected, but only peak 1 presented activity (Figure 1C). The SDS-PAGE analysis of peak 1 (Figure 1D) evidenced two bands, 62 and 33 kDa, both under reducing and non-reducing conditions. Western blot analysis (Figure 1E) showed that both were recognized by Anti-Atroxlysin-III (SVMP-III from *B. atrox* venom). The 33 kDa band was only present in peak 2 (Figure 1E). We deduced that it would be a process of autoproteolysis. An additional step using an MPLC system was conducted to separate the complete active enzyme from the proteolyzed fragments. A homogeneous 62 kDa band was obtained, which was reduced to a 53 kDa band after PNGase F treatment. (Figure 1F) In addition, Pic-III represents almost 22% of all transcripts in fresh venom (Figure 1G). This amount is not significantly different to Bpic-LAAO, but it is lower than PLA2 and higher than Pictobin, which are important toxic components of the *B. pictus* venom [27,28].

### 3.2. Functional Characterization of Pictolysin-III

The pH and temperature dependences of the enzymatic activity of Pic-III were measured (Figure 2). Values of similar proteolytic activity higher than 10 azocasein units were observed at pH 6.0 to 10.0, being the maximum value close to pH 8.0, with a drastic fall of activity at pH lower than 6.0 (Figure 2A). Pic-III exhibited high activity below 25 °C, while maintaining moderate activity between 35 °C and 55 °C. At 75 °C, the enzyme lost 80% of its proteolytic activity (Figure 2B). Likewise, the hydrolysis of azocasein by Pic-III was moderately inhibited by the reducing agent 2β-mercaptoethanol (47%) and marimastat (45%) but drastically inhibited by DTT (100%) and the chelating agent EDTA (95.8%). In contrast, the enzyme was not affected by PMSF (Figure 2C). The pre-incubation of Pic-III with Ca^2+^ and Mg^2+^ cations increased the proteolytic activity but Zn^2+^ decreased it significantly (Figure 2C). Additionally, the neutralization of Pic-III was evaluated using half, one, and two neutralizing doses of polyvalent antibotropic serum (PAS) and the residual activity was 72, 63, and 53%, respectively (Figure 2D). We evaluated if the proteolytic activity of Pic-III on azocasein depended on glycosylation. As Figure 2E,F shows, the enzymatic activity decreased to approximately 40% after its deglycosylation with PNGase F.

### 3.3. Interaction with a TLE (Pictobin) and Plasma Inhibitor α2-Macroglobulin (α2-M)

The α2-M is a human plasma inhibitor that interacts and blocks virtually any protease [51], including some snake toxins, as we previously reported [28]. Therefore, we evaluated whether α2-M inhibited the proteolytic activity of Pic-III and Pictobin, a thrombin-like enzyme isolated from *B. pictus* venom [28]. Pic-III was significantly inhibited by α2-M (50%), but this inhibition was reduced when Pic-III was co-incubated with Pictobin and α2-M (only 20% of enzymatic inhibition). No significant changes in the azocasein hydrolysis were observed when only Pic-III and Pictobin were pre-incubated. Little or no activity upon the substrate was observed with only Pictobin or α2-M, respectively. These results suggest that there is a greater affinity between Pictobin and α2-M than for Pic-III, demonstrating a possible functional synergy between both toxins.

### 3.4. Pictolysin-III Acts as a GPVI Antagonist That Produces Hemorrhage In Vivo

**Figure 3:**
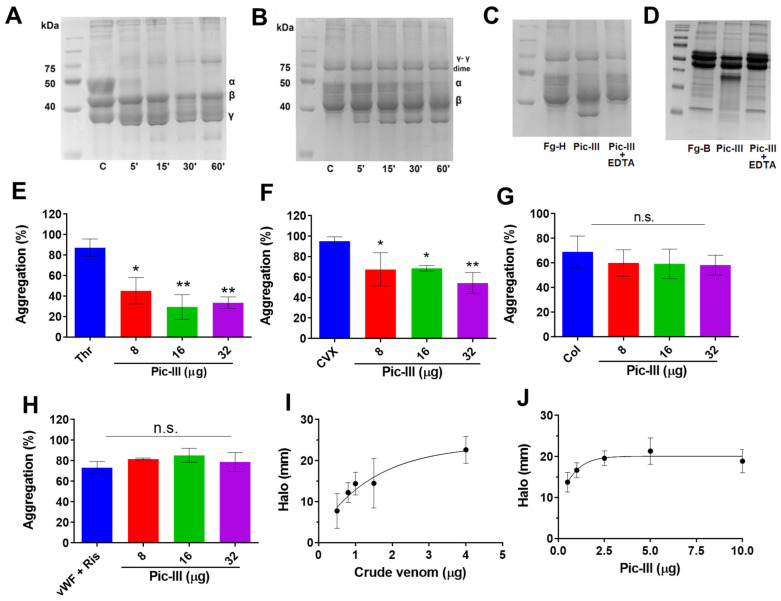
Biological activities of Pictolysin-III. Digestion of (**A**) H-Fg and (**B**) fibrin. Both digestion reactions were conducted using 1 µg of Pic-III and were analyzed with 14% SDS-PAGE. The lane description of gels is C: control; time digestion: 5-, 15-, 30-, and 60-min. Polypeptide chains of H-Fg control (α, β, and γ) and fibrin control (γ-γ dimer, α, and β) are indicated at the right. Pic-III degrades human (**C**) and bovine (**D**) fibrinogen in the same way. In both cases, the activity is significantly inhibited by the chelating agent EDTA. Effect of Pic-III on human platelet aggregation stimulated by thrombin (Thr, 1 U/mL) (**E**), convulxin (CVX, 6 µg/mL) (**F**), collagen-I (CoI, 10 μg/mL) (**G**), von Willebrand factor (vWF, 5.5 μg/mL) plus ristocetin (0.5 mg/mL) (**H**). Platelet aggregation was recorded by aggregometry. (**I**,**J**) Hemorrhagic activity of *B. pictus* venom and Pic-III. Injection of PBS was used as a control. The data shown are the mean ± SD of three independent experiments. * *p* < 0.05, ** *p* < 0.01 vs. CVX or Thr; n.s., not significant.

We analyzed the fibrino(geno) activity of Pic-III on H-Fg (plasminogen-free) and fibrin (Figure 3). Under our experimental conditions, Pic-III digested the α chains of H-Fg and fibrin in a time-dependent manner at 37 °C (Figure 3A,B). No significant alterations in the electrophoretic mobility of the Bβ and γ chains of H-Fg were detected even at 60 min incubation period. Likewise, the effect of EDTA on H-Fg (Figure 3C) and bovine fibrinogen (Figure 3D) activity of Pic-III was evaluated. In both cases, the pattern of hydrolysis of the Aα chain was inhibited by the chelating agent. Since SVMP toxins act by provoking hemorrhage and affect hemostasis and thrombosis [8], the effect of Pic-III on platelet aggregation stimulated by different agonists was evaluated. For this, platelets were pre-treated for 3 min with increasing concentrations of Pic-III. We observed that the enzyme inhibited the thrombin (Thr)- and convulxin (CVX)- induced aggregation (Figure 3E,F) but did not interfere with collagen (Col)- and von Willebrand Factor + ristocetin (vWF + Ris)-induced aggregation (Figure 3G,H). In addition, *B. pictus* venom and Pic-III showed hemorrhagic activity in vivo with a MHD = 0.6 µg and 0.3 μg, respectively (Figure 3I,J).

### 3.5. Structural Characterization of Pictolysin-III

**Figure 4:**
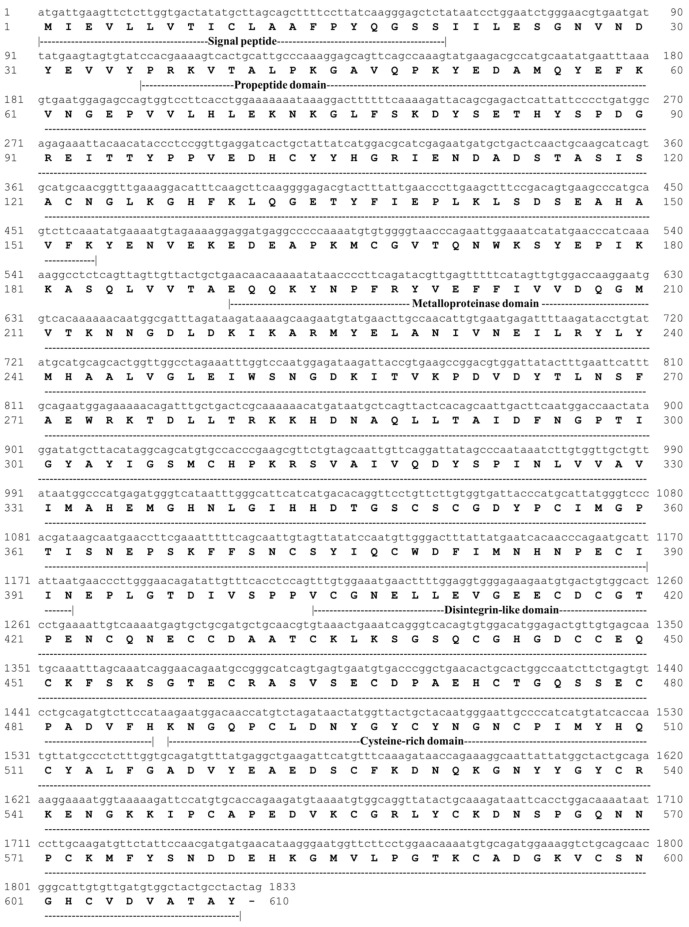
The cDNA and deduced amino acid sequence of Pictolysin-III. The cDNA sequence with 1830 pb encodes an open reading frame for 610 amino acid residues. The deduced amino acid sequence is comprised of signal peptide (1–20), pro-peptide domain (26–153), metalloproteinase domain (190–392), disintegrin-like domain (404–486), and cysteine-rich domain (487–610). The deduced amino acid sequence is represented by one-letter code.

**Figure 5:**
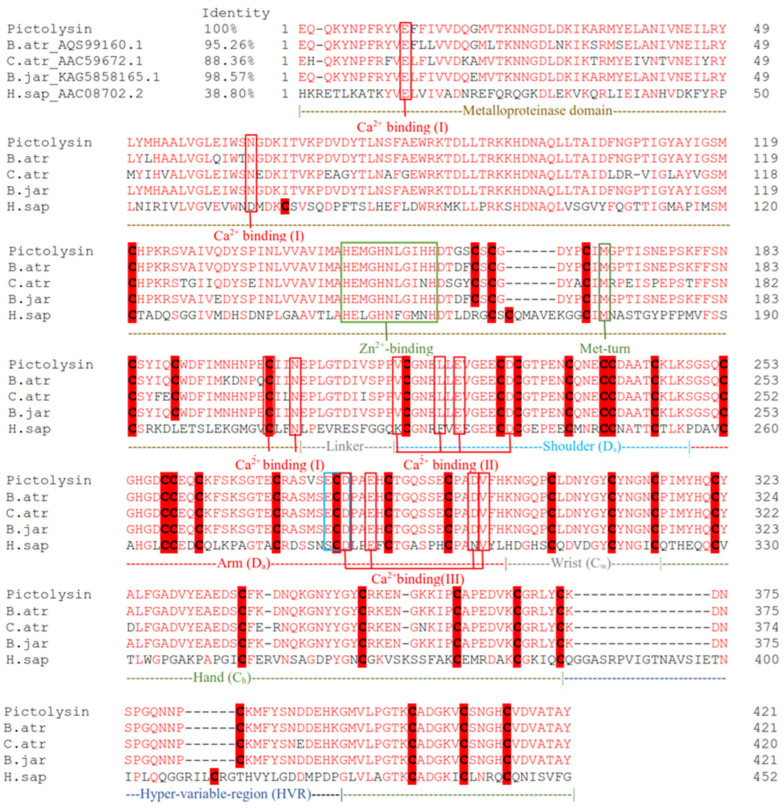
Multiple alignment of Pictolysin-III primary structure with SVMP-III homologs from bothropic venoms and human ADAM. The conserved residues are colored red and the cysteine residues are shaded in red. The hypervariable region (HVR), Ca^2+^ binding sites, and Zn^2+^ binding sites are boxed in blue, red, and green, respectively. The M domain, D domain (D_s_ and D_a_), and C domain (C_w_ and C_h_) are drawn schematically. Abbreviations: B.atr: *Bothrops atrox*, C.atr: *Crotalus atrox*, B.jar: *Bothrops jararaca*, H.sap: *Homo sapiens*.

**Figure 6:**
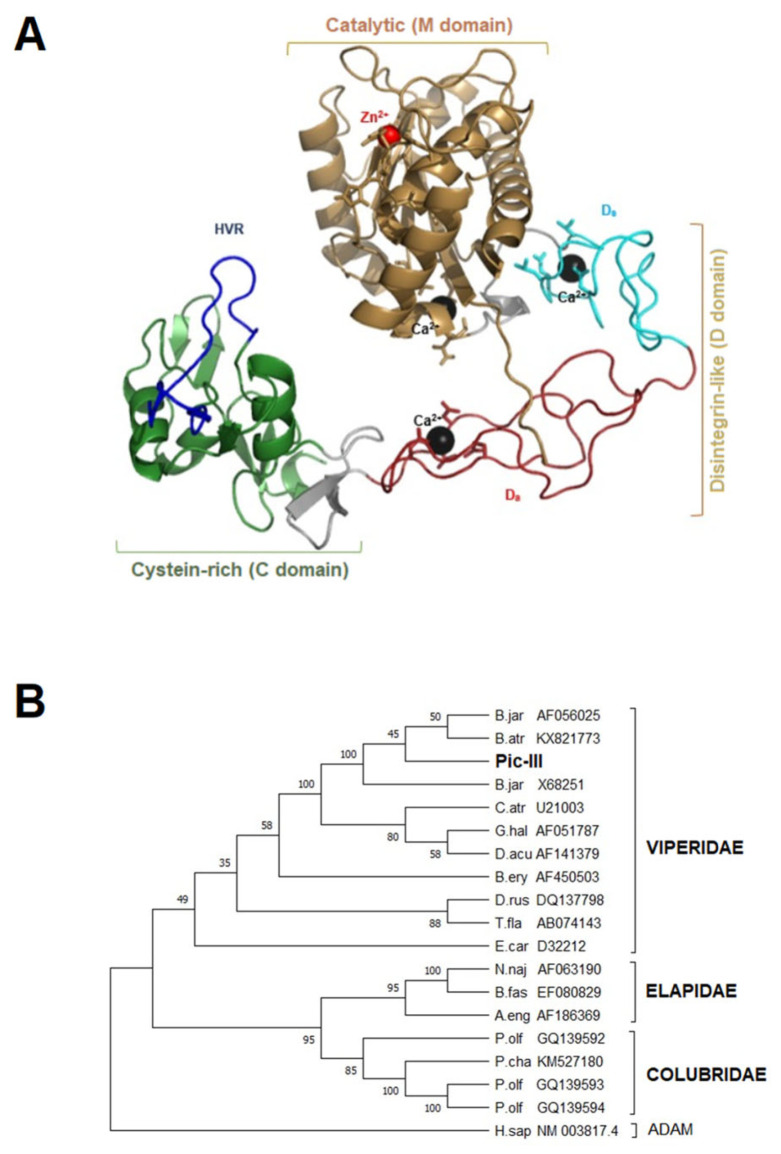
Prediction of the structural model and evolutionary relationship for Pictolysin-III. (**A**) The three-dimensional structure of protein reveals the presence of three domains: M, D, and C. The M domain at the N-terminal (yellow sand, 1–203) has a Zn^2+^-binding site (145-HEMGHNLGIHH-155) and a Ca^2+^-binding site (E12, N203, D96, and C200). A linker sequence lies between the M and D domains (gray, 204–214). The D domain (215–298) could be divided into two subdomains, the “shoulder” (D_s_-domain) (cyan, 215–248) and the “arm” (D_a_-domain) (red, 249–298). The D domain presents two Ca^2+^-binding sites, one in the D_s_-domain (V215, L220, E222, and D228) and other in the D_a_-domain (D279, D294, E282, and V295). Another linker sequence lies between the D and C domains (gray, 299–315). The C domain (green, 316–421) at the C-terminal presents the hypervariable region (blue, 373–394). The binding sites were graphed as sticks. (**B**) Phylogenetic tree of P-III SVMPs based on nucleotidic sequences. ADAM 7 from *H. sapiens* was used as an out-group. Bootstrap values are shown at each node. Abbreviations: B.jar: *Bothrops jararaca*, B.atr: *Bothrops atrox*, C.atr: *Crotalus atrox*, D.acu: *Deinagkistrodon acutus*, B.ery: *Bothrops erythromelas*, D.rus: *Daboia russelii*, T.fla: *Trimeresurus flavoviridis*, E.car: *Echis carinatus*, N.naj: *Naja naja*, B.fas: *Bungarus fasciatus*, A.eng: *Atractaspis engaddensis*, P. olf: *Philodryas olfersii*, P. cha: *Philodryas chamissonis*, H.sap: *Homo sapiens*.

The primary sequence of Pic-III has 610 amino acid residues and presents a signal peptide (1–20) and pro-peptide domain (26–153). The predicted functional sequence consists of 421 amino acid residues and presents a metalloproteinase domain (M domain), a disintegrin-like domain (D domain), and a cysteine-rich domain (C domain) (Figure 4). The predicted functional sequence shows a theoretical pI = 5.13 and Mr = 46771.7 Da and also shows 35 cysteine residues. The Pic-III architecture is similar to other SVMP P-III members of the Reprolysin family (Figure 5). The Ca^2+^-binding sites are conserved and are found in the M and D domains. Likewise, the canonical zinc-binding motif (HEXXHXXGXXH) and Met-turn are found in the D domain of Pic-III. The alignment also shows that Pic-III exhibits the cysteine residues conserved in other SVMP P-III toxins. The predicted three-dimensional structure of Pic-III harbors the classical MDC domain architecture (metalloproteinase domain, disintregin-like, cysteine-rich) (Figure 6A and Supplementary Figure S1), structure prototype of mammalian ADAM proteins [40]. In the N-terminal region, Pic-III presents a globular M domain, followed by the D domain, which is divided into two structurally distinct subsegments, the “shoulder” segment (Ds) and the “arm” segment (Da). The C domain, which is in the C-terminal region, has the “hypervariable region” (HVR). Moreover, the phylogenetic tree revealed that Pic-III is evolutionary close to other SVMP P-III from the Viperidae species, which form a distinct clade to the two composed of Elapidae and some rear-fanged species [52,53] (Figure 6B). Finally, an N-glycosylation site was found in Asn183.

### 3.6. Pictolysin-III Promotes Actin Network Disruption, Reducing the Viability of MDA-MB-231 and RMF-621 Cells

**Figure 7:**
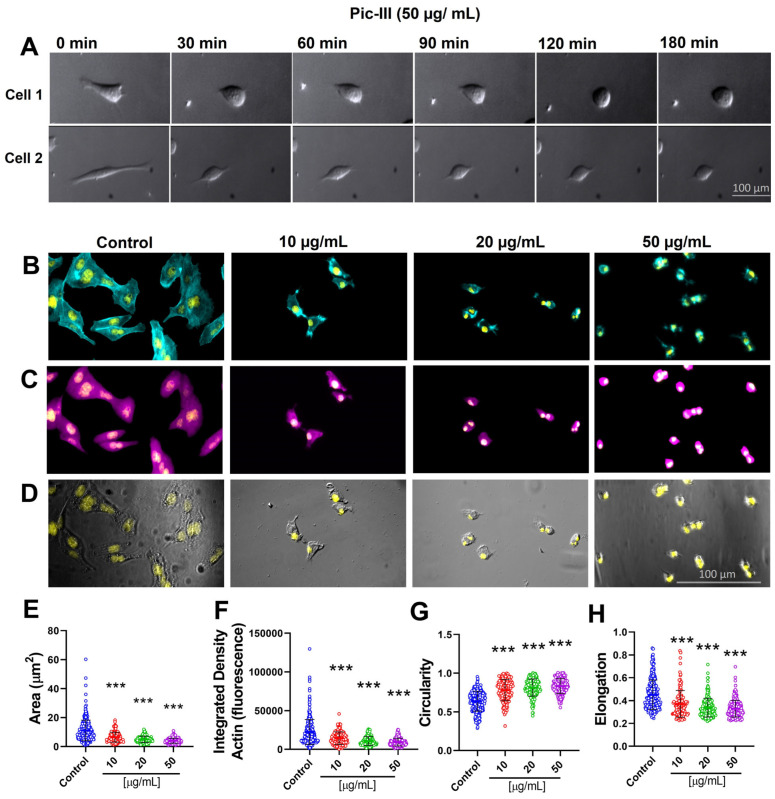
Pictolysin-III modifies Caco-2 cell morphology. (**A**) Representative images of a time-lapse experiment of Caco-2 cells treated with 50 µg/mL Pic-III during 3 h with DIC microscopy. (**B**) Representative images of Caco-2 cell treatments were DAPI (yellow), rhodamine phalloidin (cyan), (**C**) Mask Deep Red (magenta), and (**D**) DIC (white). (**E**) Quantification of morphological changes in Caco-2 cells, respectively, treated with 10, 20, and 50 µg/mL Pic-III during 3 h (*n* = 329; 143; 193; 229 independent cells, respectively). Changes in spreading cell (area), (**F**) density of F-actin, (**G**) circularity (values range 1 round), and (**H**) elongation (normalized ratio of the square of the perimeter by the area). The data shown are mean ± SD. *** *p* < 0.001 vs. control.

To determine the effect of Pic-III on the morphology of cell lines, a time-lapse experiment was conducted. As Figure 7A shows, before treatment with Pic-III, Caco-2 cells exhibited projections of the plasma membrane with an epithelial morphology. At three hours of treatment, Pic-III blocked the formation of cellular projections (Figure 7A), producing disruptions in the actin network (Figure 7B–D) and reducing the cell spreading (Figure 7E). These morphological changes were characterized by an aggregation of actin (an increased density of F-actin, Figure 7F), increased circularity (values were close to 1 in the presence of the Pic-III treatments, Figure 7G), and loss of cell polarization (reduction of elongation, Figure 7H) in a concentration-dependent manner.

**Figure 8:**
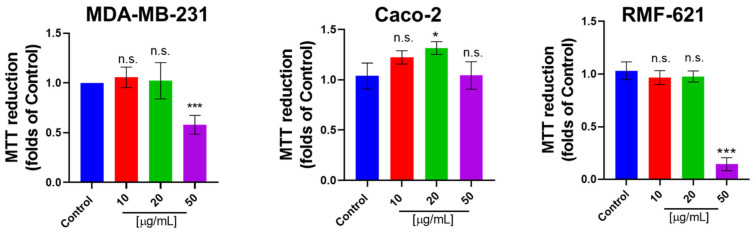
Effect of Pic-III on the MTT reduction in epithelial (MDA-MB-231 and Caco-2) and stromal (RMF-621) cells. Cells were treated with Pic-III (10, 20, and 50 μg/mL) for 48 h and cell viability was determined by MTT assay. Data are expressed as means ± SD. * *p* < 0.05; *** *p* < 0.001 vs. control; n.s., not significant.

Since the ECM–cell interaction is relevant to adapt the metabolic requirement for promoting cell viability [22], we evaluated the effect of Pic-III on the viability in epithelial (MDA-MB-231 and Caco-2) and stromal (RMF-621) cells. Our results indicate that Pic-III reduces the viability in MDA-MB-231 and RMF-621 cells at 50 µg/mL and Caco-2 cells were unaffected (Figure 8).

### 3.7. Pictolysin-III Inhibits the Basal and Maximal Mitochondrial Respiration Cell Lines

**Figure 9:**
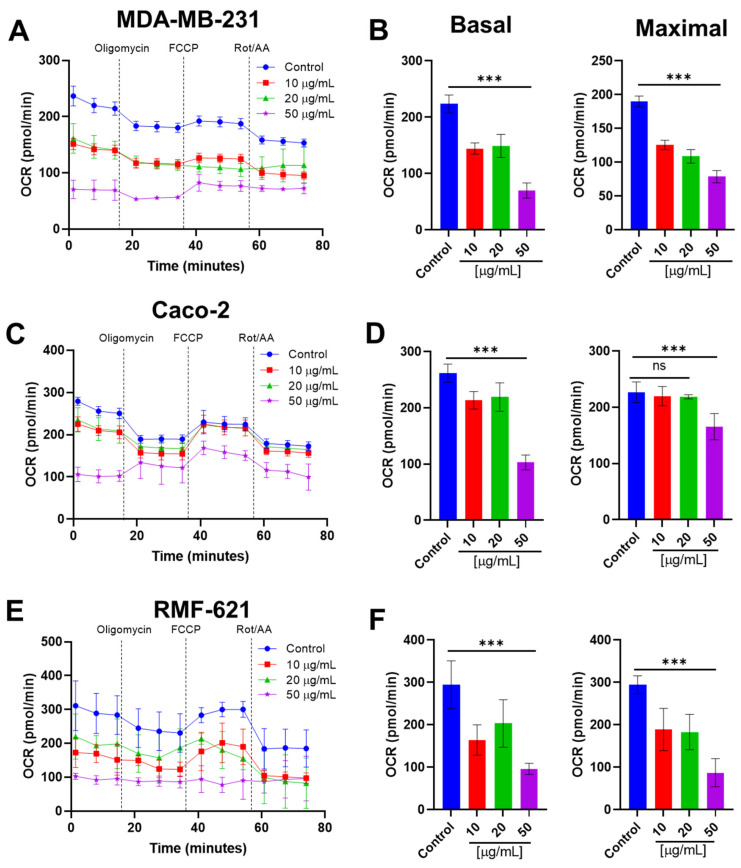
Pic-III reduces mitochondrial respiration. (**A**,**C**,**E**) Changes in the profile of respiration of MDA-MB-231, Caco-2, and RMF-621 cells induced by Pic-III. (**B**,**D**,**F**) Changes in the basal and maximal OCR in MDA-MB-231, Caco-2, and RMF-621 cells, respectively, treated with 10, 20, and 50 µg/mL Pic-III. Rot: Rotenone; AA: Antimycin A. Data are expressed as means ± SD. *** *p* < 0.001 vs. control; n.s., not significant.

Based on previous results, we evaluated the effect of Pic-III on mitochondrial respiration and glycolysis in MDA-MB-231, Caco-2, and RMF-621 cells, using real-time measurements of oxygen consumption rate (OCR) and extracellular acidification rate (ECAR), respectively. As Figure 9 shows, changes in the profile of respiration of MDA-MB-231, Caco-2, and RMF-621 were observed when stimulated with Pic-III for 8 h. MDA-MB-231 and RMF-621 cells decreased the basal OCR and maximal OCR (which represent the maximal electron transport flux) in a concentration-dependent manner (Figure 9B,F). In contrast, Caco-2 cells decreased the basal OCR in all concentrations of Pic-III; however, maximal OCR was only reduced at 50 μg/mL (Figure 9C,D). Therefore, these results indicate that Pic-III decreases the electron transport chain in MDA-MB-231, Caco-2, and RMF-621 cells, an event previously unreported for some SVMP-III. Notably, 50 µg/mL Pic-III completely reduced the ATP-linked respiration (oligomycin-sensitive OCR) in RMF-621, and it stimulated the proton-leak-driven respiration in Caco-2 cells. In MDA-MB-231 cells, this concentration of Pic-III lacked an effect on oligomycin-sensitive respiration, suggesting differential effects on OXPHOS coupling in the three cell lines (Figure 9A,C,E). Notably, non-mitochondrial OCR was reduced in the three cell lines evaluated, suggesting that other cellular oxidative reactions not linked to energy metabolism may be inhibited by Pic-III (Supplementary Figure S2).

### 3.8. Pictolysin-III Reduces Glycolysis in MDA-MB-231 and RMF-621, but Increases It in Caco-2 Cells

**Figure 10:**
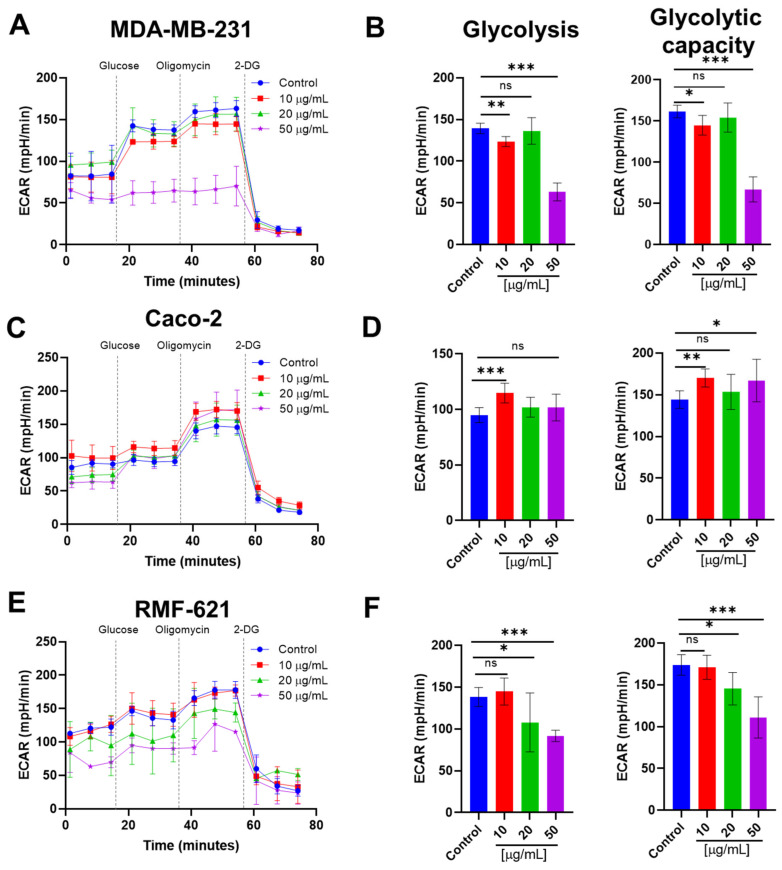
Changes in glycolysis induced by Pic-III. (**A**,**C**,**E**) Changes in the profile of extracellular acidification rate (ECAR) of MDA-MB-231, Caco-2, and RMF-621 cells induced by Pic-III. (**B**,**D**,**F**) Effect of Pic-III on glycolysis and glycolytic capacity in MDA-MB-231, Caco-2, and RMF-621 cells, respectively. 2-DG: 2-deoxy-D-glucose. Data are expressed as means ± SD. * *p* < 0.05, ** *p* < 0.01, *** *p* < 0.001, vs. control; n.s., not significant.

Inhibition of mitochondrial respiration and, consequently, the ATP synthesis, promotes a metabolic shift toward glycolysis. We speculate that the inhibitory effect of mitochondrial respiration induced by Pic-III may promote a compensatory increase in glycolysis to maintain the intracellular ATP levels as described [54]. After 8 h of treatment, effects on glycolysis were different between the three cell lines (Figure 10). RMF-621 and MDA-MB-231 cells reduced the glycolysis and glycolytic capacity at 50 μg/mL Pic-III (Figure 10A,B,E,F). No changes in the glycolytic reserve were observed (Supplementary Figure S3). On the other hand, Caco-2 cells exhibited an increase in glycolysis, suggesting a possible adaptive metabolic shift (Figure 10C,D). Collectively, these results suggest that Pic-III differentially affects glycolysis in the three cell lines.

### 3.9. Pictolysin-III Alters the Intracellular ATP, NAD(P)H, and Mitochondrial ROS Levels

**Figure 11:**
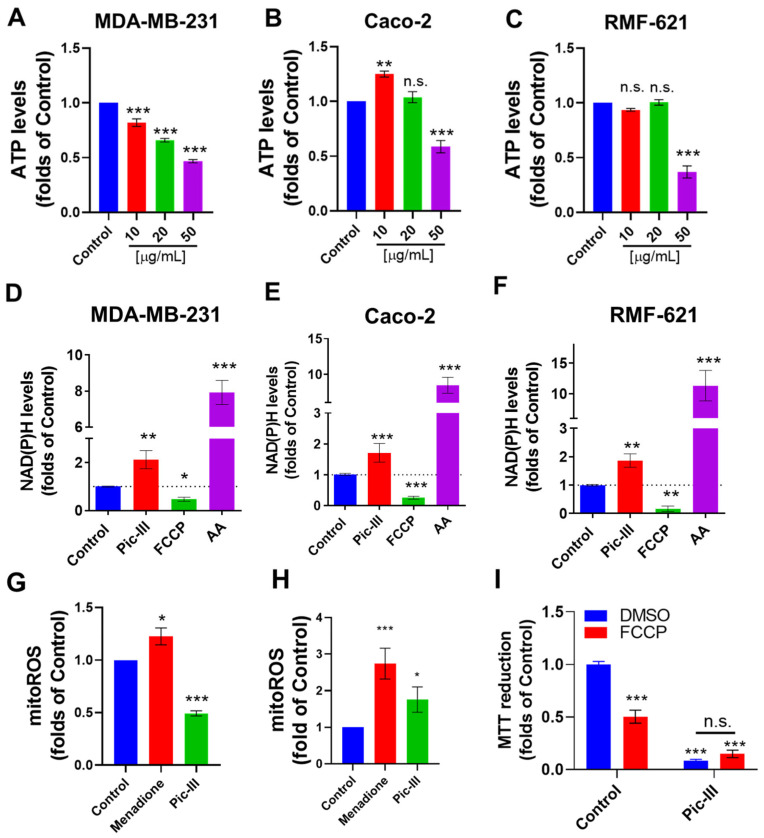
Pic-III reduces the intracellular ATP levels and affects the NAD(P)H and mitochondrial ROS. (**A**–**C**) Effect of Pic-III on ATP levels. (**D**–**F**) Effect of Pic-III (50 µg/mL), antimycin A (5 µM), and FCCP (5 µM) on NAD(P)H levels in MDA-MB-231, Caco-2, and RMF-621 cells. (**G**) Effect of Pic-III (50 µg/mL) on mitochondrial ROS in RMF-621 cells and (**H**) Caco-2 cells at 48 h, (**I**) Effect of the protonophoric agent FCCP (1 µM) on the inhibitory effect of the metabolic capacity of Pic-III (50 µg/mL) in RMF-621 cells. Data are expressed as means ± SD. * *p* < 0.05, ** *p* < 0.01, *** *p* < 0.001 vs. control; n.s., not significant.

To evaluate if the mitochondrial respiration and glycolysis inhibition by Pic-III promotes a metabolic dysfunction, we evaluated the effect of this toxin on the intracellular ATP, NAD(P)H, and mitochondrial ROS (mtROS) levels. Pic-III reduced the intracellular ATP levels in the three cell lines (Figure 11A–C), with MDA-MB-231 cells more affected in a concentration-dependent manner (Figure 11A). In Caco-2 cells, a significant increase in ATP levels was observed at 10 µg/mL (Figure 11B), which correlated with the metabolic remodeling toward enhanced glycolysis (Figure 10D).

Since inhibition of maximal OCR produced by Pic-III might imply a reduction of respiratory complexes activity [55], producing a disruption in the NAD/NADH ratio, we evaluated the effect of Pic-III on NAD(P)H in the epithelial and stromal cell lines. As Figure 11D–F shows, FCCP (a protonophore agent that uncouples OXPHOS) and antimycin A (a respiratory complex III inhibitor) produce a decrease and increase of NAD(P)H levels, respectively, and Pic-III (50 µg/mL) significantly increases the NAD(P)H close to two folds of the control in the three cell lines.

The respiratory complex I contributes about 40% of the proton motive force required for mitochondrial ATP synthesis, through the oxidation of NADH and producing superoxide [56,57]. Interestingly, mtROS levels were reduced in RMF-621 cells, but in Caco-2 cells, mtROS was increased after 48 h of treatment (Figure 11G,H). Finally, we evaluated whether mitochondrial membrane potential (Δψm) is required for the effect of Pic-III. For this, RMF-621 cells were pre-treated with FCCP (1 µM) for 1 h and then, were exposed to Pic-III for 48 h (Figure 11I). FCCP reduced the viability to 0.50 ± 0.06 folds of the control (*p* < 0.001 vs. control), Pic-III to 0.09 ± 0.01 folds of the control (*p* < 0.001 vs. control), and a combination of FCCP + Pic-III to 0.15 ± 0.03 folds of the control (*p* < 0.001 vs. control). No significant differences were observed between Pic-III treatment and FCCP + Pic-III, suggesting that Δψm is not a determinant of the effect of Pic-III. Collectively, these results suggest that Pic-III reduces cellular metabolism, affecting energy production, and being more active in MDA-MB-231 breast cancer cells.

### 3.10. Pictolysin-III Increases the Secretion of Cytokines in Caco-2 and RMF-621 Cells

**Figure 12:**
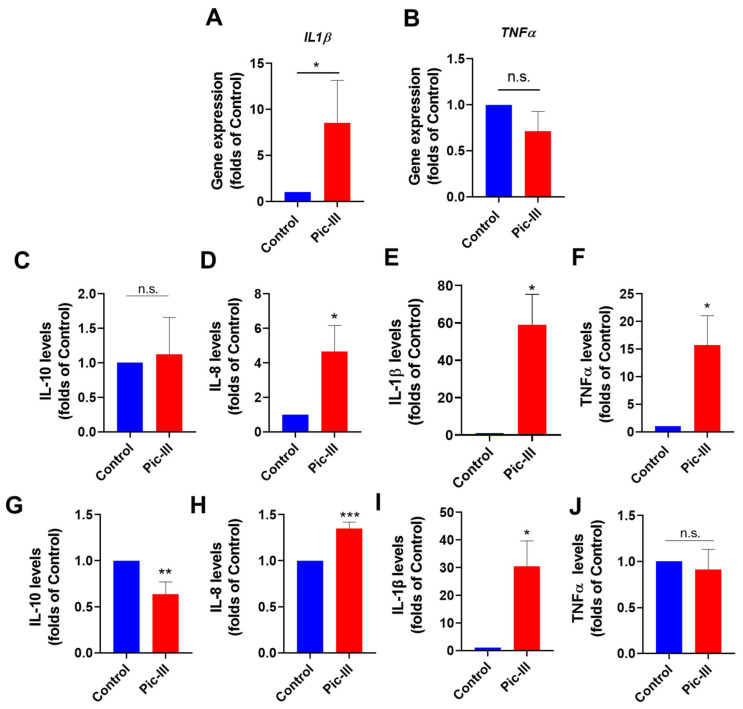
Pic-III promotes the secretion of cytokines. (**A**,**B**) Effect of Pic-III on the expression of IL1β and TNFα genes in RMF-621 cells treated for 8 h, (**C**–**F**) Effect of Pic-III on cytokine production in RMF-621 and (**G**–**J**) Caco-2 cells. Cells were treated with Pic-III for 48 h with non-cytotoxic concentrations (RMF-621 cells: 20 µg/mL and Caco-2 cells: 50 µg/mL) and secreted cytokine levels (IL-10, IL-8, IL-1β, and TNFα) in the culture medium were determined by CBA assay. Data are expressed as means ± SD. * *p* < 0.05, ** *p* < 0.01, *** *p* < 0.001 vs. control; n.s., not significant.

Previously, we described that fibroblast RMF-621 under metabolic stress conditions changes the expression of pro-inflammatory genes [58]. Based on this, the effect of Pic-III on the expression of IL1β and TNFα genes was evaluated. At 8 h of treatment, Pic-III increased the mRNA levels for IL1β without effects on TNFα gene expression (Figure 12A,B). As it has been recognized that mitochondrial dysfunction can promote the secretion of cytokines [59,60,61], we determined the effect of Pic-III on cytokine secretion in RMF-621 and Caco-2 cells at non-cytotoxic concentrations for 48 h. Pic-III generated changes in the cytokine secretion profile of RMF-621 cells, increasing the IL-8, IL-1β, and TNF levels with no changes in IL-10 secretion (Figure 12C–F). Pic-III induced the production of IL-8 and IL-1β, decreasing IL-10 in Caco-2 cells (Figure 12G–I). TNFα was not induced by Pic-III in this cell line (Figure 12J).

### 3.11. Pictolysin-III Produces Sensitization to BH3 Mimetic ABT-199 (Venetoclax) in MDA-MB-231 Cells

**Figure 13:**
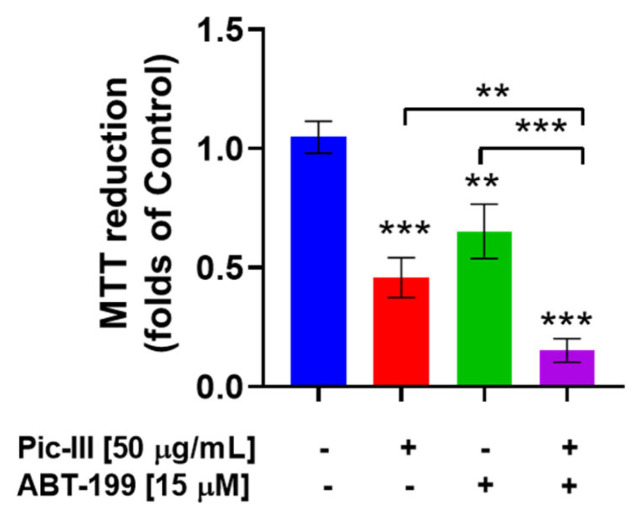
Pic-III sensitizes to the BH3 mimetic drug ABT-199 in MDA-MB-231 cancer cells. Effect of Pic-III (50 µg/mL), ABT-199 (15 µM), and combination Pic-III + ABT-199 on the viability of MDA-MB-231 at 48 h of exposition. Data are expressed as means ± SD. ** *p* < 0.01, *** *p* < 0.001 vs. control.

The inhibition of mitochondrial bioenergetics promotes an early induction of intrinsic pathway (mitochondrial pathway) apoptosis by ABT-199 [62], a BCL2-selective inhibitor with cytotoxic action in several cancer cells [63], including triple-negative breast cancer cells [64]. Therefore, we speculated that disruption of mitochondrial respiration by Pic-III may sensitize the cytotoxic effect of ABT-199. As Figure 13 shows, ABT-199 and Pic III reduced the viability to 0.65 ± 0.11 (*p* < 0.01 vs. control) and 0.45 ± 0.08 folds of the control (*p* < 0.001 vs. control), respectively, at 48 h of treatment. Notably, the combination Pic-III + ABT-199 reduced the viability to 0.15 ± 0.05 folds of the control (*p* < 0.001 vs. control), suggesting that Pic-III-induced mitochondrial dysfunction produces sensitization to BH3 mimetic ABT-199 (Venetoclax) in MDA-MB-231 cells.

## 4. Discussion

Snake venom metalloproteinases (SVMPs) are the primary factors responsible for snake-venom-induced local and systemic hemorrhage [8,43]. Particularly, the largest SVMPs, categorized into the P-III class, present a high structural complexity associated with a high functional diversity of their M, D, and C domains [17]. In this work, we describe the purification and characterization of Pictolysin-III (Pic-III), a novel hemorrhagic P-III metalloproteinase isolated from *B. pictus*, affecting mitochondrial bioenergetics of cell lines. Our results can contribute valuable knowledge to the structure–function relationship of this protein family, and the recently explored mechanisms of venom on cancer biology.

Our purification protocol shows that Pic-III undergoes autoproteolysis (Figure 1D). This event previously reported for other SVMPs could occur due to alkaline conditions, Ca^2+^ absence, and slightly elevated temperature promoting autolysis between the M and DC domains. Although the presence of other proteases that promote the separation of these domains that are removed during the purification process cannot be ruled out [65].

Pic-III has a proteolytic activity dependent on the pH and temperature, which was optimal at pH 8.0 and 25 °C, respectively. These values are close to the experimental data obtained in other SVMP-III such as leucurolysin [66] and bothropoidin [42]. Interestingly, it was observed that at lower temperatures (5 °C and 10 °C), the proteolytic activity was high. Possibly, this is because autoproteolysis is lower at these temperatures, maintaining more enzyme units than at physiological temperature. We regard this fact as an exclusive event of the in vitro condition and not representative of an enzymatic characteristic involved in the biological action of Pic-III. In the presence of Ca^2+^ and Mg^2+^, Pic-III significantly increases its proteolytic activity in line with SVMPs [30,42,67].

SVMPs generally exhibit fibrin(ogen)olytic and extracellular-matrix-degrading (hemorrhagic) activities [68]. Pic-III is a venom alfa fibrin(ogen)olytic enzyme that breaks down fibrin clots and avoids clot formation by degrading fibrinogen, actions which allow the spreading of venom compounds [69] and alteration of the homeostatic system [70]. In addition, it has been well-documented that many SVMPs inhibit platelet aggregation induced by different agonists [71]. Pic-III does not affect the collagen- or vWF-induced aggregation but inhibits aggregation induced by thrombin and CVX, a very potent GPVI agonist isolated from rattlesnake (*Crotalus durissus terrificus*) venom [72,73]. Collagen induces platelet aggregation by interacting with GPVI and α2β1 integrins, whereas vWF binds to the GPIb complex [74,75]. Our results suggest that Pic-III acts as a GPVI antagonist but does not interfere with the GPIb pathway. On the other hand, human α2-M is a plasmatic inhibitor blocking the active site of many SVMPs and some serineproteases such as Pictobin (thrombin-like enzyme from *B. pictus*) [28,76,77]. Since a greater affinity between Pictobin and α2-M than for Pic-III was observed in our experiments, a potential Pic-III–Pictobin synergistic interaction may be involved in the hemorrhagic effect of *Bothrops pictus* venom [6]. The gene structure of all SVMP presents a signal peptide and a pro-peptide domain before the M domain, cleaved by proteolytic processing before being secreted from snake venom glands [78]. Mature SVMP P-III has thirty-five strictly conserved cysteine residues among the Reprolysin family with three, eight, and six disulfide bridges formed in the M, D, and C domains, respectively [78]. However, a cysteine residue (Cys189 of Pictolysin-III) does not form a disulfide bridge because its side chain is embedded in the hydrophobic core and only is conserved in SVMP P-III.

Pic-III contains a protein motif conserved in zinc-dependent metalloproteases (HEXXHXXGXXH), where histidine residues play a fundamental role in the interactions with cofactors for enzymatic activity [79]. Moreover, this conserved sequence and the Met-turn are characteristics of members of the metzincins [80]. The disintegrin-like domain contains a motif that interacts with integrins: ECD, a characteristic region of SVMP P-III and ADAM proteins, that replaces the RGD domain of the disintegrins [81]. The predicted molecular weight of Pic-III (46771.7 Da) can be different from SDS-PAGE due to potential protein glycosylation. Although the molecular basis is not understood, SVMP P-III is more hemorrhagic and tends to have long carbohydrate chains [82]. The predicted glycosylation of Pic-III (Asn183) was close to the methionine turn, whose extrinsic flexibility is proposed as a critical factor in the hemorrhage [82,83]. Similar to other snake proteases (e.g., [28,29,30]), our results demonstrate that Pic-III activity is dependent on N-linked carbohydrates, as its activity was reduced by up to 45% after deglycosylation with PNGase F treatment.

Alternatively, Pic-III contains all the calcium-binding residues for three calcium ions: site I in the M domain, which is coordinated by oxygen atoms from the side chain of Glu12, Asn203, and Asp96, and the carbonyl group of Cys200; site II in the D domain, coordinated by oxygen atoms from Val215, Leu220, Glu222, and Asp228 residues; and site III, also in the D domain, which is coordinated by oxygen atoms of Asp279 (from the ECD motif), Glu282, Asp294, and Val 295 [84].

The predicted model of Pictolysin-III presents the classic architecture of the MDC domains (metalloproteinase, disintegrin-like, and cysteine-rich domains), a structure that is the prototype of mammalian ADAM proteins [40]. The M domain shows an α/β scaffold forming four helices (H1, H2, H3, and H4), a five-stranded parallel β-sheet (strands S1, S2, S3, S4, and S6), and an anti-parallel strand (S5), a type of secondary structure corroborated in other SVMPs P-III [40,84]. This domain is separated from the DC domains with the sequence EPLGTDIISP [85] and has a loop conformation in Pic-III. The Ds segment protrudes from the M domain, opposes the catalytic site, and is close to the Ca^2+^-binding site [40]. Domain D together with domain C, form a “C”-shape with its surface concave towards domain M. This structure is maintained by cysteine residues and Ca^2+^-binding residues, which are strictly conserved in most ADAM proteins [78].

Mitochondrion-driven innate immunity involves the ability of mitochondrial DAMPs release (e.g., mtDNA, cardiolipin, formyl-methionine-labeled peptides, and cytochrome c) to activate pattern recognition receptors (e.g., Toll-like receptors) and trigger a pro-inflammatory cascade [86,87,88]. Recently, it has been shown that the venom of *B. laceolatus* releases mtDAMPs from cardiomyocytes, and in a murine model, *B. asper* venom induces the release of mtDNA and cytochrome c in the circulation [89], suggesting that mitochondria would mediate inflammatory signals in the envenomed tissue environment [89,90,91]. Notably, the inhibition of mitochondrial respiration stimulated by glutamate plus malate (complex I’s substrates), succinate (complex II’s substrate), and TMPD (an artificial complex IV’s substrate) plus ascorbate is a previous event for mtDAMPs production [90]; however, the toxin classes involved remain uncertain. Accordingly, the crude venom of viper *Macrovipera lebetina obtusa* affects respiration in the Vero monkey kidney epithelial cell line, with this effect sensitive to metalloproteinase and phospholipase inhibitors [21]. To our knowledge, there are no studies that identify the action of an isolated SVMP on mitochondrial respiration and its implication for the disruptive action of the cell–ECM interaction.

Our results suggest that Pic-III produces morphological changes in cells, decreases lamellipodia formation, and induces actin network disruption, reducing mitochondrial bioenergetics in epithelial (MDA-MB-231 and Caco-2) and stromal (RMF-621 fibroblast) cell lines. It has recently been described that some PLA2 from snake venom can interact with respiratory complexes, inhibiting their activity [92]; however, we have not been able to establish whether Pic-III interacts directly with any respiratory complex in the mitochondrion. In MDA-MB-231 and RMF-621 cells, Pic-III blocked the glycolysis, with the former cell line more affected, damaging the ability to remodel the metabolism for maintaining ATP levels. Under mitochondrial respiration inhibition, mtROS production can drive pro-inflammatory cytokine production [61,93] and trigger NLRP3 inflammasome activation [94]. Although it has been extensively reported that SVMPs induce the secretion of pro-inflammatory mediators [95,96], our results suggest an unexpected link between mitochondrial respiration inhibition and IL-1β production by Pic-III in epithelial and stromal cells that requires further studies.

Mitochondrial bioenergetics is driven by ECM composition for supporting the initial steps of metastasis and chemoresistance [22,97]. The ECM–cancer-cell interaction requires the ability of these cells to adhere to ECM components and migrate through them [98]. Integrins activate intracellular signaling controlling cytoskeleton organization, cell polarity, leading-edge formation, and mitochondrial bioenergetics of migrating cancer cells [22,99,100]; therefore, disrupting the ECM–integrin–metabolism axis is an attractive anti-cancer target for new antagonist molecules based on SVMP scaffolds. Accordingly, some SVMPs P-III inhibit proliferation [101] and induce apoptosis [102,103] in cancer cells. Interestingly, our results indicate that the triple-negative breast cancer (TNBC) cell line MDA-MB-231, which exhibits high metabolic plasticity for supporting migration [45,104], was more sensitive to the Pic-III treatment, reducing ATP levels and mitochondrial respiration compared to breast fibroblast RMF-621.

The BCL-2-selective inhibitor ABT-199 (Venetoclax) is a potent and orally bioavailable anti-cancer drug in several cancer cells [63], including TNBC cells [64]. In leukemic cells, ABT-199 has high efficacy in inducing apoptosis in nanomolar and sub-micromolar concentrations [62]; however, it induces intrinsic apoptosis at higher concentrations in breast cancer cells [105]. Reports suggest that *IDH*1/2 mutations [106], inhibition of glutaminolysis [107], and mitochondrial cristae remodeling [108], and OXPHOS uncoupling [62] increase the anti-cancer efficacy of ABT-199. Notably, our results indicate that Pic-III sensitizes to ABT-199 in MDA-MB-231 cells, which may be mediated by inhibition of the mitochondrial respiration. Nonetheless, the cell signaling triggered by Pic-III-induced mitochondrial dysfunction in cancer cells remains to be elucidated. Finally, Pic-III is the first of the SVMPs reported with action on mitochondrial bioenergetics. Emergent evidence suggests that several snake venom toxin classes (e.g., phospholipases [92,109], thrombin-like enzymes [28], three-finger toxins [110,111]) act on the mitochondrial bioenergetics [3], exhibiting unique and novel mechanisms that are not yet fully understood. This highlights the mitochondrion as an essential player in the snake venom action that requires more studies.

## 5. Conclusions

We characterize a novel type-III snake venom metalloprotease, called Pictolysin-III (Pic-III), isolated from *Bothrops pictus* venom. Pic-III is a 62.5 kDa proteinase and has an optimum temperature and pH of 40 °C and 7.5, respectively. It hydrolyzes dimethyl casein, azocasein, gelatin, fibrinogen, and fibrin. In human platelets, it inhibits the CVX- and thrombin-induced platelet aggregation, acting as a GPVI antagonist and in vivo, exhibits hemorrhagic action. Notably, Pic-III induces actin network disruption, reduces the mitochondrial respiration and ATP levels in epithelial (MDA-MB-231 and Caco-2) and stromal (RMF-621) cells, promoting pro-inflammatory cytokine secretion and sensibilization to the cytotoxic BH3 mimetic ABT-199 (Venetoclax). Pic-III is the first SVMP reported with action on mitochondrial bioenergetics. Importantly, since several SVMPs are promising lead compounds that inhibit platelet or ECM–cancer-cell interactions, it is necessary to further understand their mechanisms of action to reduce undesirable toxic effects.

## Data Availability

Not applicable.

## Supplementary Materials

The following supporting information can be downloaded at: https://www.mdpi.com/article/10.3390/pharmaceutics15051533/s1, Figure S1: Ramachandran plot: Most amino acid residues in the Pictolysin-III structure presented a favorable stereochemistry (95.6%); Figure S2: Effect of Pic-III on non-mitochondrial respiration in MDA-MB-231, Caco-2, and RMF-621 cells after 8 h of treatment; Figure S3: Effect of Pic-III on glycolytic reserve in MDA-MB-231, Caco-2, and RMF-621 cells after 8 h of treatment.
