# Supplementary material for: Pictolysin-III, a Hemorrhagic Type-III Metalloproteinase Isolated from Bothrops pictus (Serpentes: Viperidae) Venom, Reduces Mitochondrial Respiration and Induces Cytokine Secretion in Epithelial and Stromal Cell Lines
Source: Pharmaceutics. 2023 May 18;15(5):1533. doi: 10.3390/pharmaceutics15051533 (PMC10222873; doi:10.3390/pharmaceutics15051533)

SUPPLEMENTARY INFORMACION

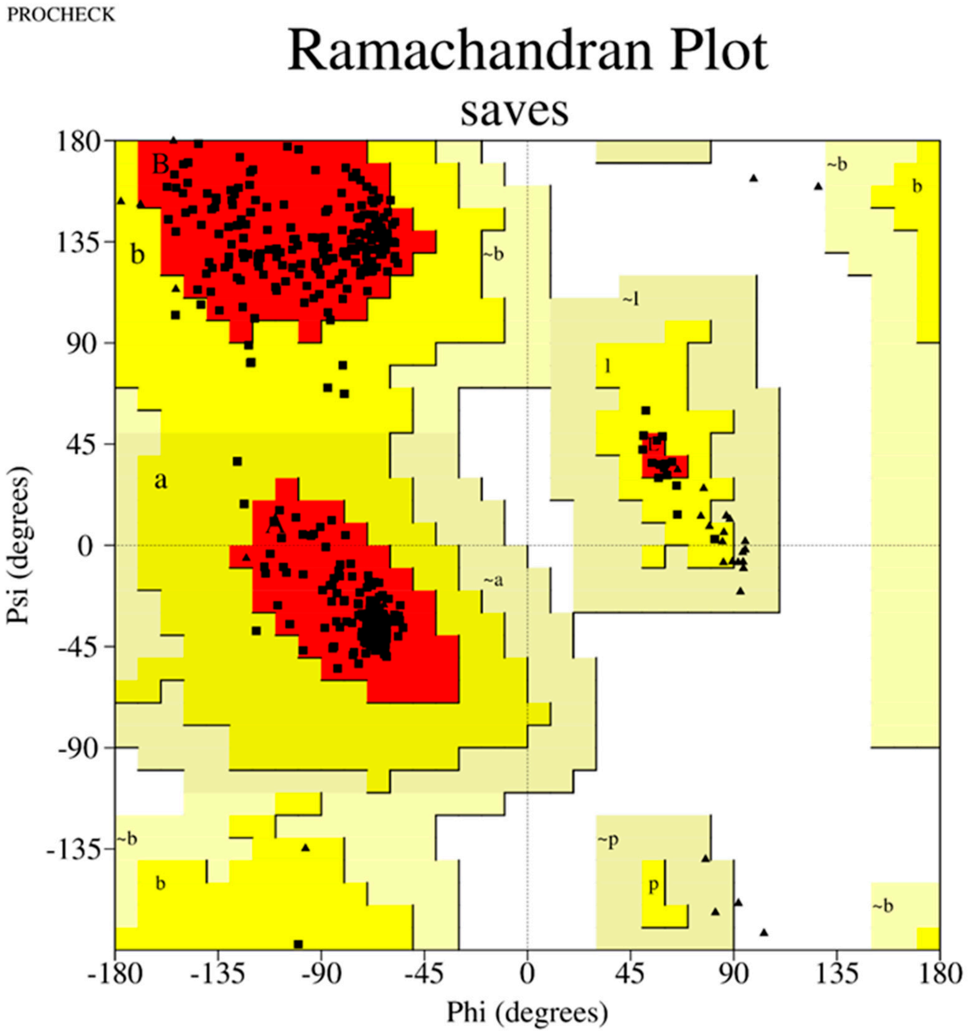

Supplementary Figure S1. Ramachandram plot: Most amino acid residues in Pictolysin III structure presented a favorable stereochemistry (95.6 %).

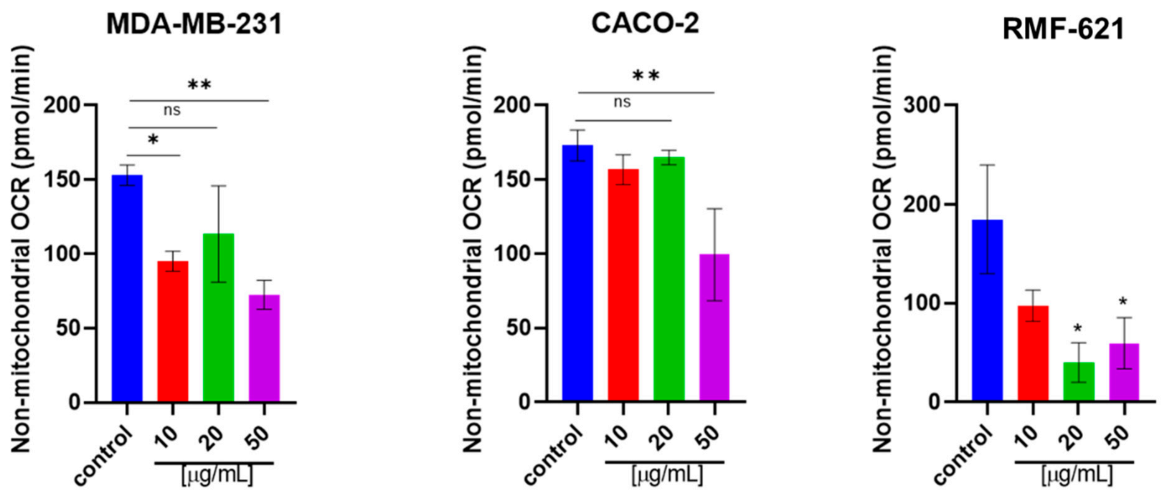

Supplementary Figure S2: Effect of Pic-III on non-mitochondrial respiration in MDA-MB-231, Caco-2, and RMF-621 cells after 8 h for treatment. Data are expressed as means ± SD. *p < 0.05, **p < 0.01, vs Control, and n.s.: not significant.

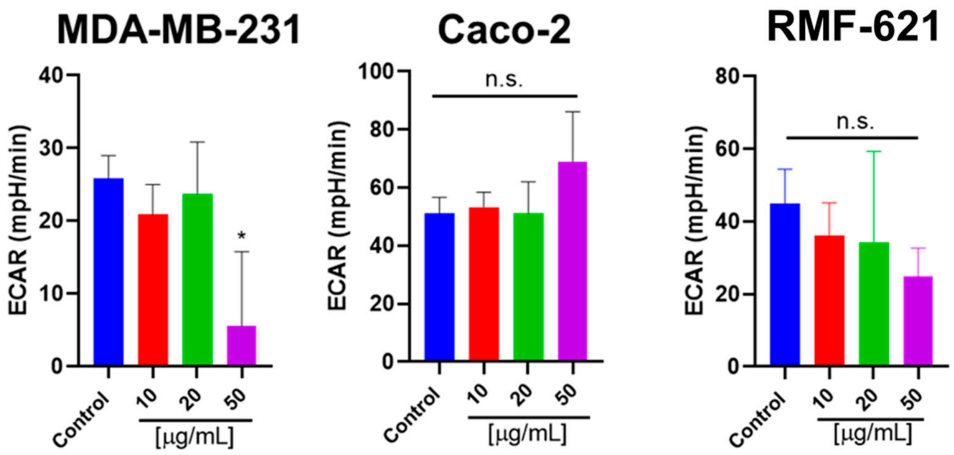

Supplementary Figure S3: Effect of Pic-III on glycolytic reserve in MDA-MB-231, Caco-2, and RMF-621 cells after 8 h for treatment. Data are expressed as means ± SD. *p < 0.05 vs Control, n.s.: not significant.

Uncropped gels

Relative to Figure 1D

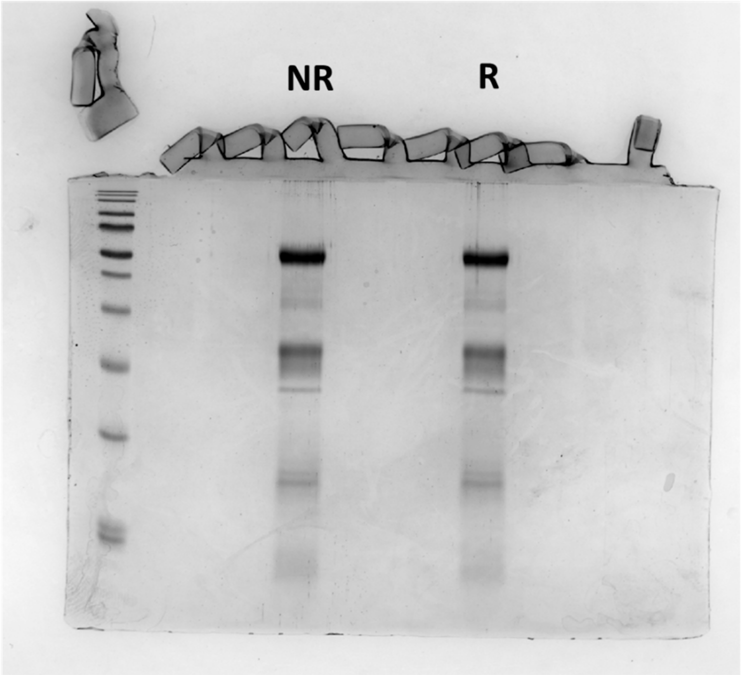

Relative to Figure 1E

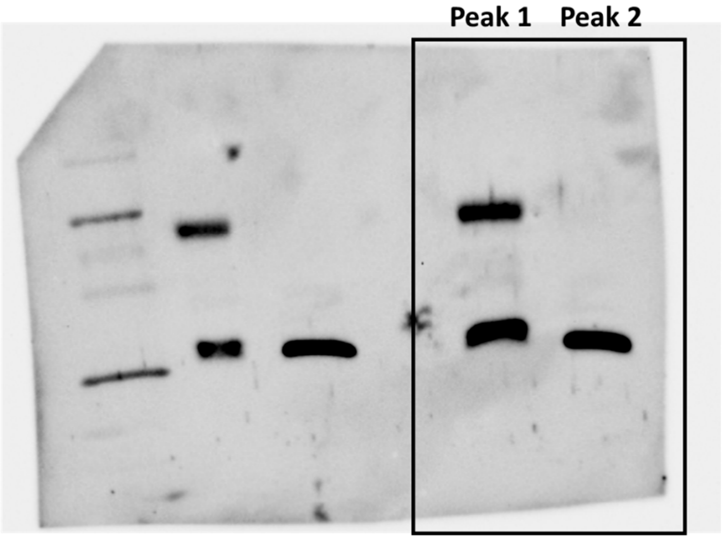

Relative to Figure 1F

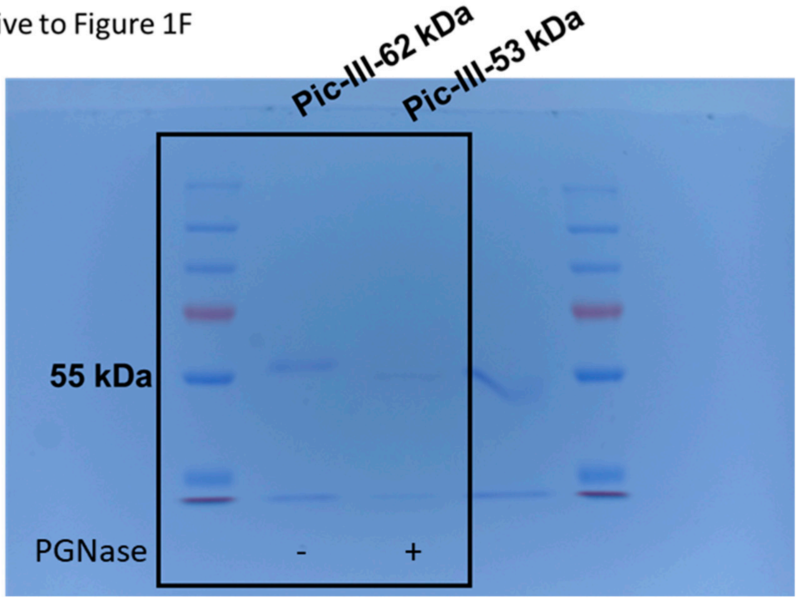

Relative to Figure 3A

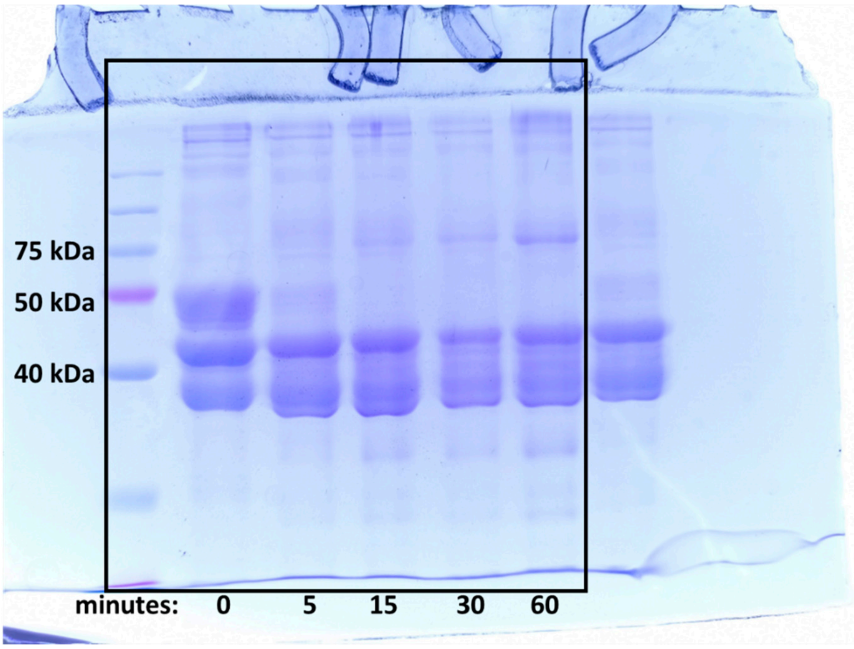

Relative to Figure 3B

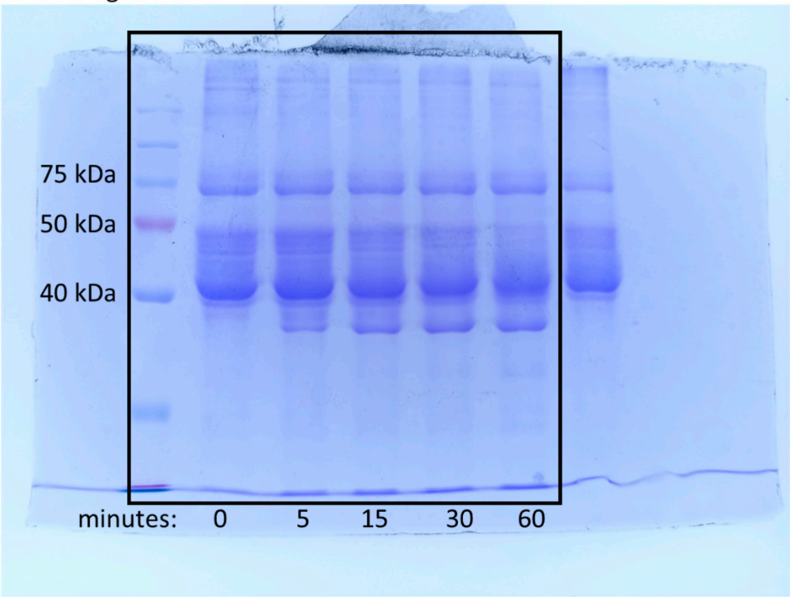

Relative to Figure 3C

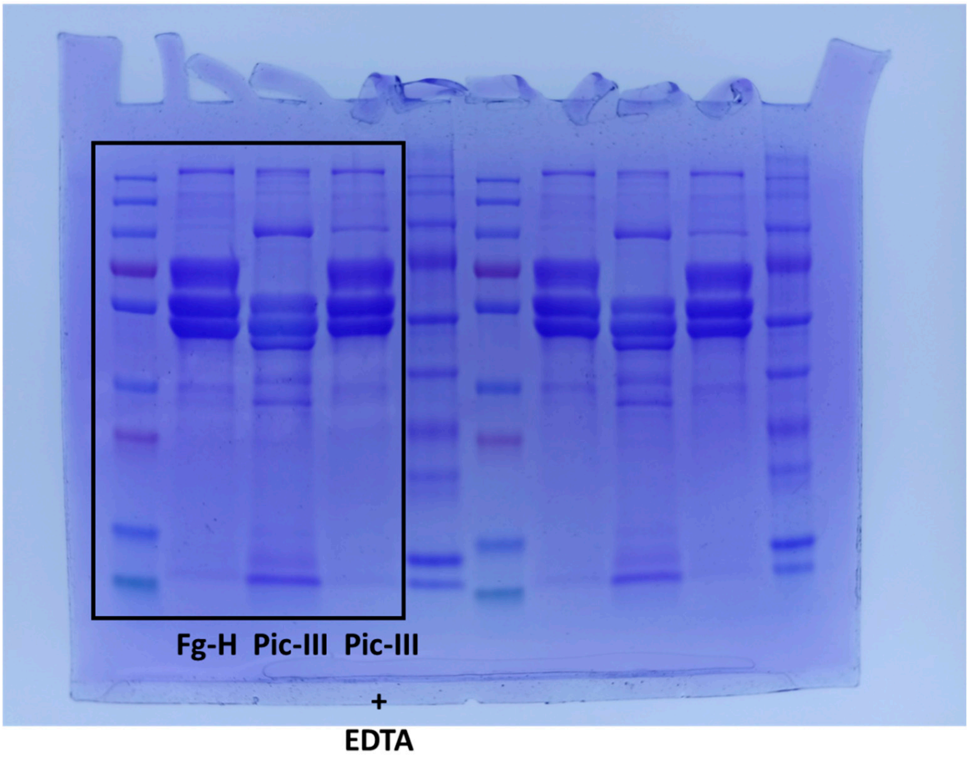

Relative to Figure 3D

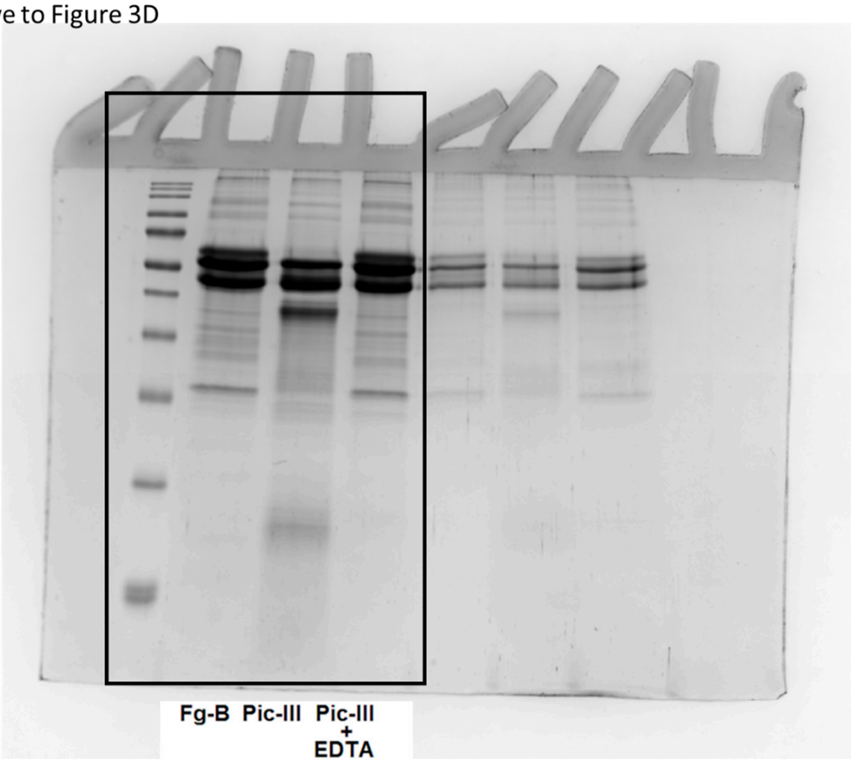

Supplement: Supplementary file 1 [file pharmaceutics-15-01533-s001.zip › pharmaceutics-2163664-supplementary.pdf]
